# *CYP2J2* Modulates Diverse Transcriptional Programs in Adult Human Cardiomyocytes

**DOI:** 10.1038/s41598-020-62174-w

**Published:** 2020-03-24

**Authors:** Eric A. Evangelista, Theresa Aliwarga, Nona Sotoodehnia, Paul N. Jensen, Barbara McKnight, Rozenn N. Lemaitre, Rheem A. Totah, Sina A. Gharib

**Affiliations:** 1 Department of Medicinal Chemistry, University of Washington School of Pharmacy, Seattle, WA USA; 2 Cardiovascular Health Research Unit, Department of Medicine, Seattle, WA USA; 3 Division of Cardiology, Department of Epidemiology, Seattle, WA USA; 4 Department of Biostatistics, University of Washington School of Public Health, Seattle, WA USA; 5 Computational Medicine Core at Center for Lung Biology, Division of Pulmonary, Critical Care and Sleep Medicine, University of Washington, Seattle, WA USA

**Keywords:** Cardiology, Cardiovascular diseases

## Abstract

CYP2J2, a member of the Cytochrome P450 family of enzymes, is the most abundant epoxygenase in the heart and has multifunctional properties including bioactivation of arachidonic acid to epoxyeicosatrienoic acids, which, in turn, have been implicated in mediating several cardiovascular conditions. Using a proteomic approach, we found that CYP2J2 expression is lower in cardiac tissue from patients with cardiomyopathy compared to controls. In order to better elucidate the complex role played by CYP2J2 in cardiac cells, we performed targeted silencing of *CYP2J2* expression in human adult ventricular cardiomyocytes and interrogated whole genome transcriptional responses. We found that knockdown of *CYP2J2* elicits widespread alterations in gene expression of ventricular cardiomyocytes and leads to the activation of a diverse repertoire of programs, including those involved in ion channel signaling, development, extracellular matrix, and metabolism. Several members of the differentially up-regulated ion channel module have well-known pathogenetic roles in cardiac dysrhythmias. By leveraging causal network and upstream regulator analysis, we identified several candidate drivers of the observed transcriptional response to *CYP2J2* silencing; these master regulators have been implicated in aberrant cardiac remodeling, heart failure, and myocyte injury and repair. Collectively, our study demonstrates that *CYP2J2* plays a central and multifaceted role in cardiomyocyte homeostasis and provides a framework for identifying critical regulators and pathways influenced by this gene in cardiovascular health and disease.

## Introduction

Cytochrome P450s (CYPs) are a superfamily of enzymes involved in a wide range of physiological processes. These heme-containing monooxygenases have been extensively studied with the aim of understanding their role in the biotransformation and clearance of drugs and toxins^1–3^. More recently, however, there is a growing interest in a subset of cytochrome P450s involved in the bioactivation of endogenous fatty acids into signaling molecules, with an emphasis on the CYP2 family^4,5^. Members of CYP2 family oxidize several polyunsaturated fatty acids (PUFAs), many of which have roles in various cell response and signal transduction pathways^4^. Delineating how these enzymes affect and interact with other molecules in cellular response cascades could be critical in understanding the mechanisms by which cells respond to environmental changes and external stressors and may identify new therapeutic targets. Among the human CYP2 enzymes, CYP2J2 is of particular interest because it is the most abundantly expressed epoxygenase in the heart with the highest presence in ventricular cardiomyocytes^6,7^.

Our understanding of the role and impact of CYP2J2 and arachidonic acid epoxide metabolites (epoxyeicosatrienoic acids, EETs) on cardiovascular health is rapidly advancing^8^. Several studies have reported on the protective effects of EETs in various disease models, particularly for their roles in diabetes and related cardiovascular disorders. Interest in CYP2J2 is focused on its role in bioactivating arachidonic acid into EETs, specifically in the heart. In addition to arachidonic acid, CYP2J2 has a wide range of substrates including several drugs, such as terfenadine and astemizole. CYP2J2 can metabolize other compounds, specifically other polyunsaturated fatty acids (PUFAs) such as docosahexaenoic acid (DHA) and eicosapentaenoic acid (EPA)^4^. Importantly, CYP2J2’s PUFA substrates and resulting metabolites are involved in various and distinct signaling pathways. EETs, which are produced intracellularly, may act as secondary messengers, although a definitive receptor in the cardiomyocyte has yet to be identified. EETs have been shown to promote angiogenesis, regulate vascular tone, protect against hypoxia re-oxygenation injury, and regulate NO synthesis^9–11^. Importantly for the cardiomyocytes health, EETs are potent activators of several ion channels including ATP sensitive K channels (K_ATP_)^12–14^ and L-type Ca channels^15,16^. CYP2J2-associated EETs can also act through the activation of a wide range of intracellular signaling pathways such as the MAPK/ERK and Akt pathways^11,17,18^.

The role of EETs in cardiovascular health further highlights the importance of CYP2J2 in the heart. Overexpression studies have shown that increased *CYP2J2* expression leads to increased EET levels and ultimately improves protection against arrhythmogenic susceptibility in animal models^19^. We have recently shown that *CYP2J2* gene expression in cardiomyocytes is altered in response to oxidative stress^20^. We found that increased reactive oxygen species prompted cardiomyocytes to up-regulate *CYP2J2* expression—a response that was mitigated by the presence of antioxidants.

Given the multifaceted function of CYP2J2, we initially determined CYP2J2 protein levels in cardiac tissue obtained from patients with cardiomyopathy and control subjects. We then undertook an unbiased approach to systematically delineate pathways modulated by this gene in adult-derived cardiomyocytes. We used siRNA to effectively silence *CYP2J2* expression and performed RNA-sequencing (RNA-seq) and bioinformatics analyses to identify *CYP2J2*-regulated transcriptional programs in human cardiomyocytes and putative targets for future mechanistic and therapeutic studies.

## Results

### CYP2J2 protein levels are lower in individuals with cardiac disease

**Figure 1:**
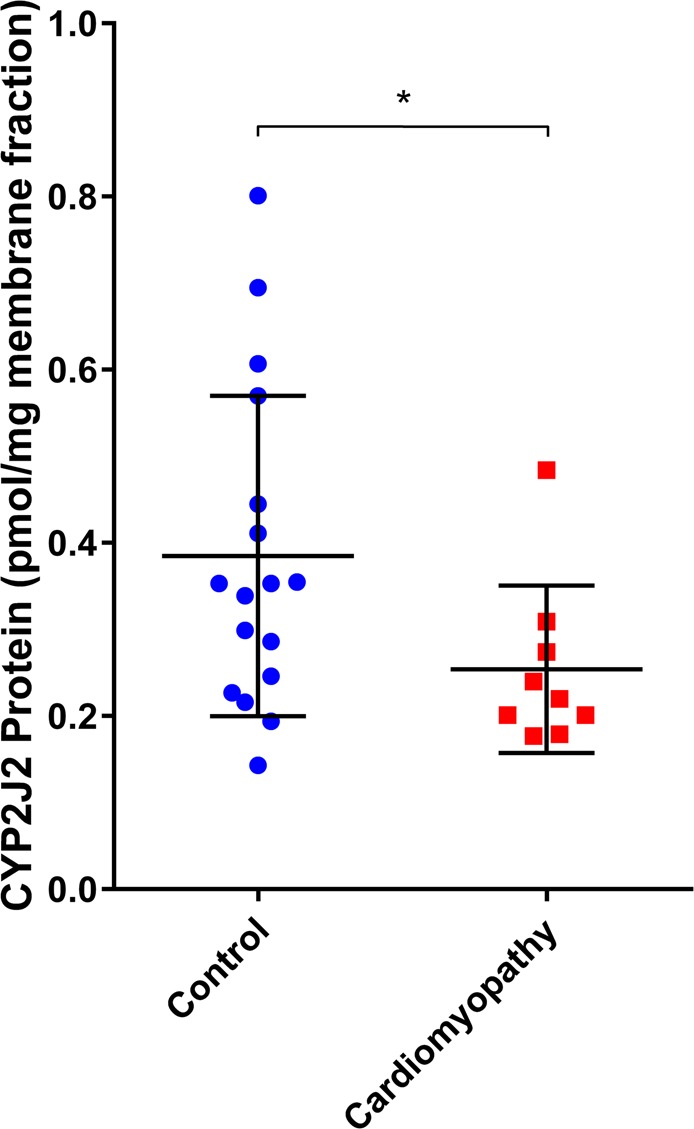
CYP2J2 protein content in ventricular tissue of Control (n = 17) and patients with cardiomyopathy (n = 9) as determined by protein mass spectrometry. CYP2J2 protein levels are significantly lower (p < 0.05) in cardiac tissues of individuals with non-ischemic cardiomyopathy compared to subjects with no known heart disease. *p < 0.05.

We utilized mass spectrometry to assess whether CYP2J2 protein content differed between individuals without cardiac disease and patients with non-ischemic cardiomyopathy. We observed significantly lower mean CYP2J2 protein levels in ventricular tissues of individuals with non-ischemic cardiomyopathy compared to ventricular tissue of subjects with no known heart disease as shown in Fig. 1.

### CYP2J2 expression knockdown results in decreased enzymatic activity

**Figure 2:**
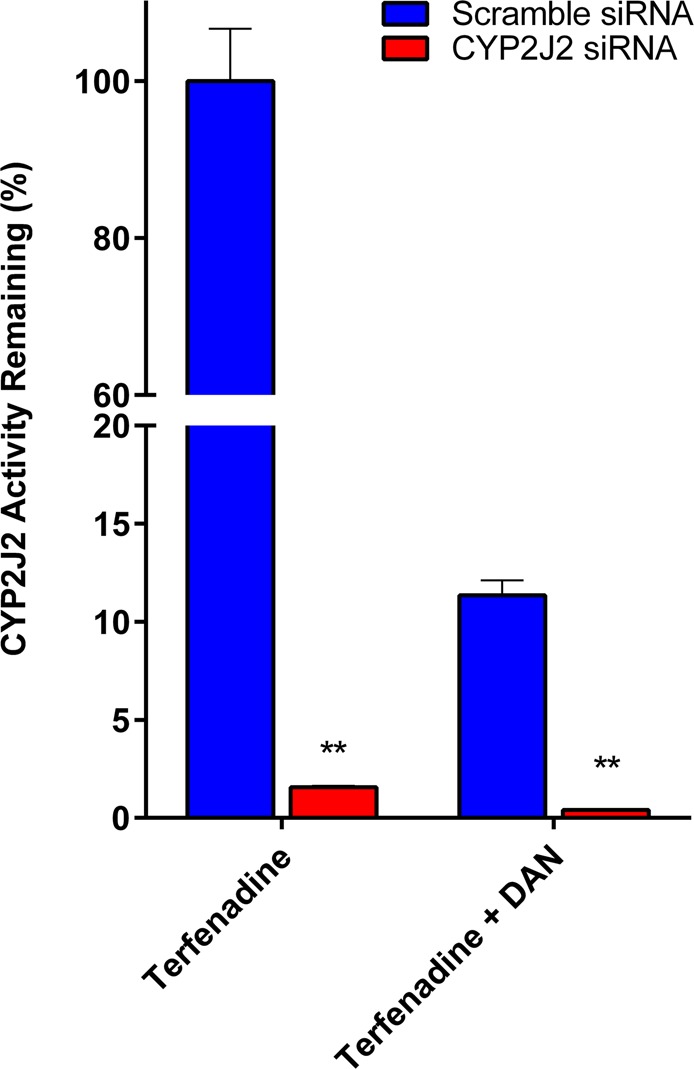
Relative mass spectrometric quantification of CYP2J2 activity in cells treated with either scrambled siRNA or CYP2J2 siRNA. Silencing with CYP2J2 siRNA for 72 hours significantly decreases (p < 0.001) CYP2J2 protein activity, as measured by terfenadine metabolism. In cells treated with CYP2J2 siRNA, activity is almost non-existent with very little hydroxyterfenadine detectable. Danazol, a known CYP2J2 inhibitor, was used to ascertain that the activity observed was due to CYP2J2, decreasing hydroxyterfenadine formation in scramble siRNA and CYP2J2 siRNA treated cells by over 90%. **p < 0.01.

To determine whether the siRNA knockdown was effective at reducing protein levels and thus activity, we measured CYP2J2 activity using terfenadine as a probe substrate. A significant decrease in terfenadine metabolism (>90%) was observed between cells treated with CYP2J2 siRNA compared to cells treated with scramble siRNA as summarized in Fig. 2. Our results also demonstrate that in the presence of a known CYP2J2 inhibitor, danazol (DAN), there is a significant drop in terfenadine hydroxylation in cells treated with siRNA, indicating that the observed changes in metabolism during siRNA treatment is due to reduction in CYP2J2 protein level and activity.

### CYP2J2 knockdown elicits widespread changes in human cardiomyocyte transcriptome

**Figure 3:**
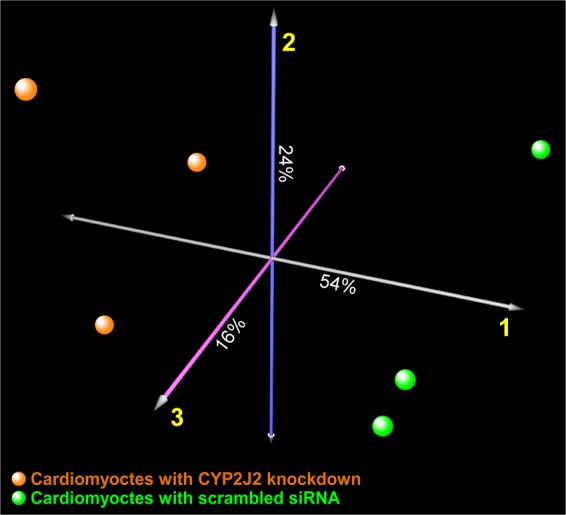
Multidimensional scaling using correspondence analysis of human cardiomyocte transcriptome in *CYP2J2*-silenced samples (n = 3, orange spheres) and scrambled siRNA controls (n = 3, green spheres) demonstrates distinct segregation between the two conditions. The percentage of the overall gene expression variability captured by each orthogonal axis is shown, with the two experimental groups separating most prominently across the first component.

**Figure 4:**
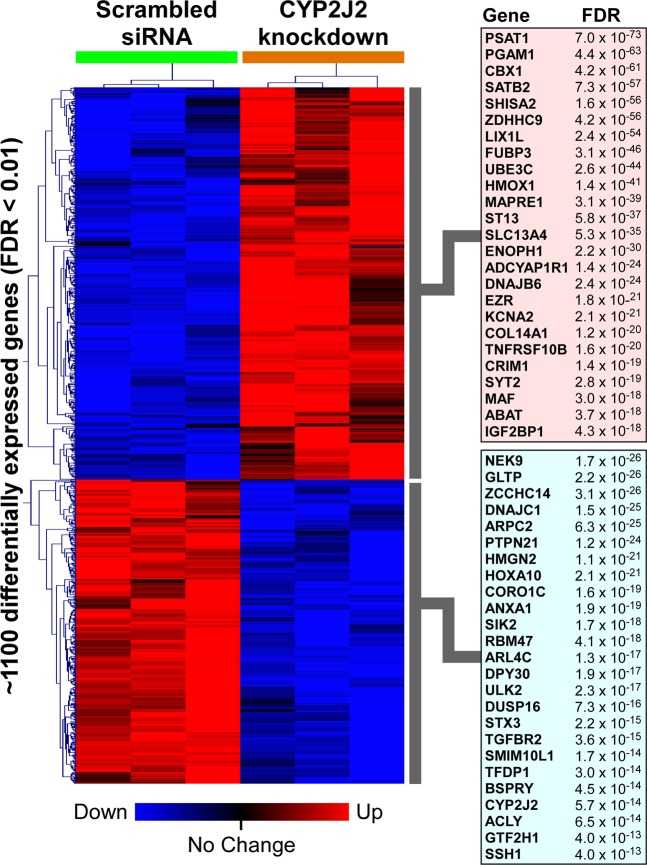
Hierarchical cluster analysis of approximately 1,100 differentially expressed genes following *CYP2J2* knockdown in human cardiomyocytes is shown as a heatmap. The top 25 up- and down-regulated genes have been labeled (please see Supplementary Table S1 for details). Note that, as expected, *CYP2J2* expression is significantly suppressed in the silenced group (FDR 5.7 × 10^−14^).

**Figure 5:**
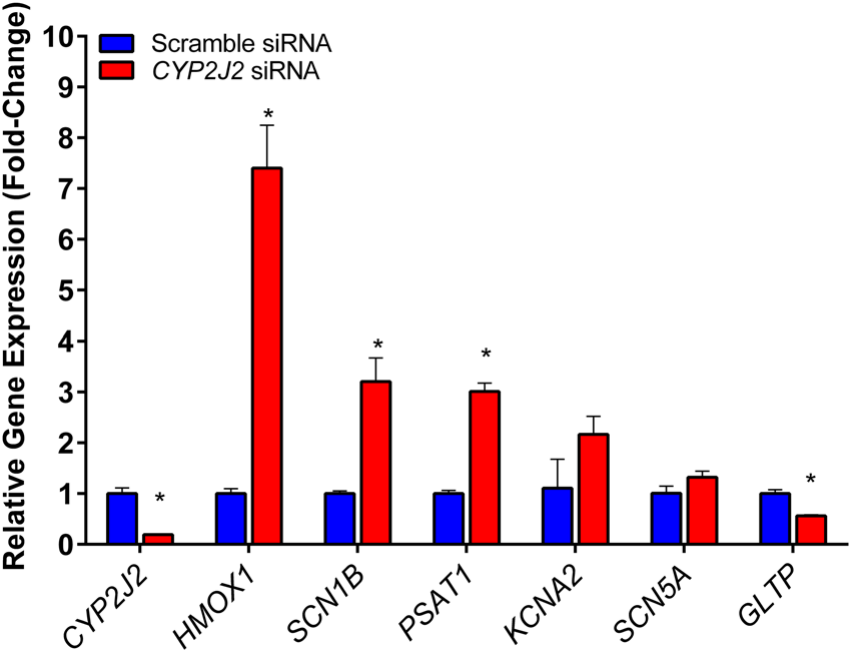
qRT-PCR quantification of a subset of genes identified as altered by RNA-seq from an independent set of experiments. *HMOX1, SCN1B, PSAT1*, and *GLTP* were significantly altered by *CYP2J2* silencing, consistent with what was observed via RNA sequencing. Additionally, *KCNA2* and *SCN5A* were up-regulated and consistent with the RNA sequencing findings, but did not reach statistical significance. *p < 0.05.

To assess the global effects of suppressing *CYP2J2* expression, we applied correspondence analysis to the entire cardiomyocyte transcriptome across the two experimental conditions. As depicted in Fig. 3, we observed clear segregation between cardiomyocytes whose *CYP2J2* was knocked down by siRNA compared to cells treated with scramble siRNA, indicating that modulating *CYP2J2* expression results in large-scale alterations in cardiomyocyte gene expression profiles. Next, by applying a strict statistical threshold (FDR < 0.01) we identified over 1,100 transcripts that were differentially expressed between *CYP2J2-*silenced cardiomyocytes vs. controls treated with scramble siRNA. Figure 4 represents these differentially expressed genes as a hierarchically clustered heatmap with the top 25 up- and down-regulated genes displayed (Supplemental Table S1). In order to validate transcriptional changes observed from the RNA-seq study, we repeated an independent set of experiments and used qRT-PCR to measure the expression of a subset of genes that were altered in response to *CYP2J2* knockdown. Figure 5 depicts the changes in the expression of *HMOX1*, *SCN1B, PSAT1, KCNA2, SCN5A*, and *GLTP* as assessed by qPCR. The first five genes had been identified as being up-regulated whereas GLTP was down-regulated based on the RNA-seq experiment and these changes were verified by qPCR.

### Highly distinct transcriptional programs are activated in cardiomyocytes following CYP2J2 silencing

**Figure 6:**
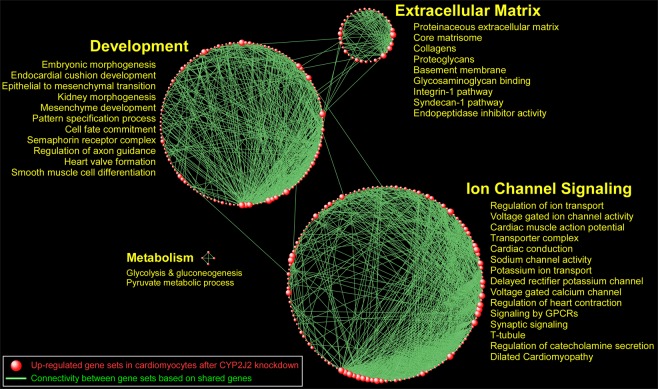
Network-based visual depiction of gene set enrichment analysis (GSEA) in adult ventricular myocytes after *CYP2J2* silencing. Each red sphere corresponds to an up-regulated gene set. Connectivity between the gene sets is based on 50% or greater overlap among their member genes. Note that the topology of the network is characterized by the emergence of biological modules comprises of highly interconnected gene sets with similar functional themes. Notable modules include “Ion Channel Signaling”, “Development”, “Extracellular Matrix”, and “Metabolism”. Representative pathways for each of these modules are shown and a complete list of enriched gene sets in included in Supplementary Table S2.

To better elucidate the transcriptional consequences of suppressing *CYP2J2* in human cardiomyocytes, we applied gene set enrichment analysis (GSEA) in order to systematically identify up and down-regulated pathways. Interrogating a large repertoire of canonical pathways and Gene Ontology categories, we observed that the predominant enrichment pattern was comprised of up-regulated processes (n = 360 gene sets, FDR < 0.05) with only one down-regulated pathway (“peptide chain elongation”, FDR = 0.005) (complete list provided in Supplemental Table S2). This finding suggests that silencing of *CYP2J2* promotes activation of a diverse set of compensatory programs. A network-based summary of the GSEA results is shown in Fig. 6, in which enriched gene sets with significant overlap of members are connected to each other, revealing larger, functionally coherent biological modules. The most prominent modules thus identified were those populated by pathways involved in “Ion Channel Signaling”, “Development”, “Extracellular Matrix”, and “Metabolism” (Supplemental Table S3).

### Inhibition of CYP2J2 expression modulates ion channel genes in cardiomyocytes

**Figure 7:**
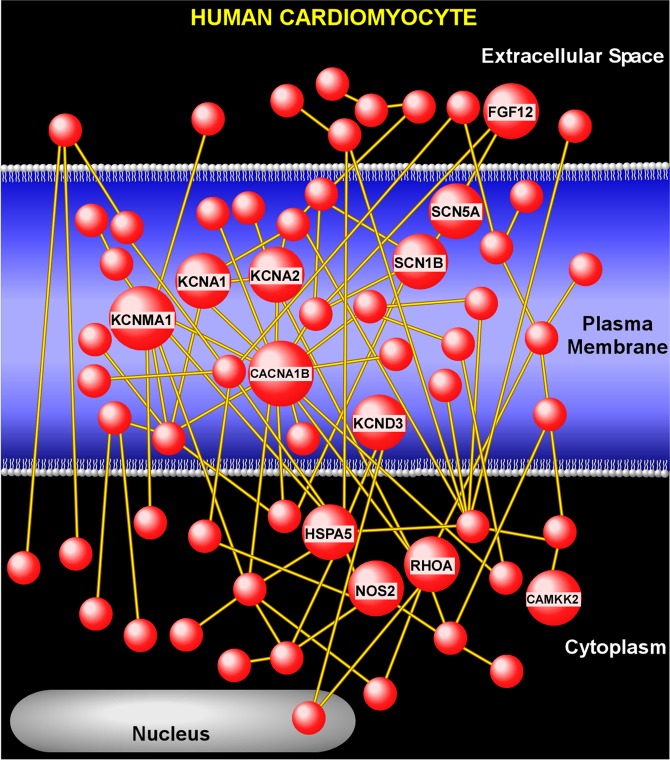
Gene product interaction network of “leading edge” members of gene sets mapping to the “Ion Channel Signaling” module. To enhance biological significance, we limited the “leading edge” genes to those with FDR < 0.01 and constructed the interaction network by incorporating only members with direct interactions. We have labeled several key nodes causing cardiac dysrhythmias including sodium (SCN1B, SCN5A), potassium (KCNA1, KCNA2, KCNMA1, KCND3), and calcium (CACNA1B) channels, as well as other genes linked to electrophysiologic abnormalities (FGF12, HSPA5, NOS2, RHOA, and CAMKK2). Full list of network nodes is provided in Supplementary Table S4.

“Ion Channel Signaling” was the largest biological module identified in our network-based gene set analysis. We further investigated this module by focusing on the subset of its genes known as the “leading edge” that includes the most significant contributors to pathway enrichment based on differential expression. We limited the leading edge genes to those differentially expressed at FDR < 0.01. We then performed network analysis to link these significantly expressed “leading edge” genes to one another based on their known direct interactions (Fig. 7, details provided in Supplemental Table S4). A number of key modulators of arrhythmogenesis and heart function were members of this network, including those associated with inherited cardiac conduction disorders such as SCN5A^21^, SCN1B^22^, KCND3^23^, FGF12^24^, as well as genes linked to electrophysiological abnormalities and cardiac dysfunction such as KCNA1^25^, KCNA2^26^, CACNA1B^27^, NOS2^28^, HSPA5^29^, RHOA^30^, and CAMKK2^31^.

### Upstream regulator analysis identifies key drivers of cardiomyocyte transcriptional response to CYP2J2 silencing

We next leveraged the unbiased nature of RNA-seq approach to identify upstream regulators by comparing the concordance between the direction of differential expression following *CYP*2J2 silencing with expected alterations in gene expression using curated knowledge bases. This analysis predicted that several master regulators of canonical pathways were activated in response to inhibition of *CYP2J2* gene expression, with the most significant being HIF1-α, Sonic Hedgehog (SHH), TGF-β, Endothelin-1 (EDN1), and β-Catenin (CTNNB1) (*P*-value range: 1.2–7.9 × 10^−6^, Supplemental Table S5). Each of these upstream regulators has been implicated in cardiac development and remodeling, heart failure, myocyte injury and repair, consistent with our findings that developmental and extracellular matrix pathways are altered following *CYP2J2* suppression (Fig. 6), and reduced protein expression of CYP2J2 in heart tissue of patients with cardiomyopathy (Fig. 1).

Collectively, our results indicate that targeting *CYP2J2* profoundly alters diverse transcriptional programs and regulatory networks in human cardiomyocytes, and that many of these molecular effects are associated with deleterious cardiovascular consequences in animal models and human-based studies.

## Discussion

In this study, we first investigated CYP2J2 protein levels using proteomic mass spectrometry in ventricular tissue obtained from a cohort of individuals without heart disease (controls) and patients with cardiomyopathy. We found that subjects with cardiac disease had lower CYP2J2 protein content than controls. This observation is consistent with emerging evidence implicating CYP2J2 as a protective enzyme in cardiac homeostasis^14,15,32–35^. We therefore sought to determine the global effects of *CYP2J2* downregulation on adult human derived ventricular myocytes using siRNA to suppress expression. We demonstrated that silencing *CYP2J2* expression profoundly decreased its enzymatic activity, and utilized this model system to further investigate the consequences of targeting the putative cardioprotective properties of CYP2J2 in the human heart.

We performed whole genome transcriptional profiling to elucidate the global effects of *CYP2J2* gene silencing in human adult-derived ventricular myocytes. A key finding of this study was the striking number of differentially altered genes and pathways following *CYP2J2* silencing, implying that this gene plays a central role in regulating cardiomyocyte homeostasis. *CYP2J2* has been demonstrated to have an important role in cardiovascular conditions, specifically as a protective enzyme against adverse cardiac events due to EET formation from arachidonic acid. However, our findings provide evidence for a more multidimensional function of *CYP2J2* in cardiac health beyond EET formation.

By leveraging pathway-based analyses, we identified a wide repertoire of processes influenced by *CYP2J2*, including those involved in “Ion Channel Signaling”, “Development”, “Extracellular Matrix”, and “Metabolism”. In the context of heart function and disease, changes in these broad categories are significant. Cardiac rhythmogenesis, for example, is due to sodium, potassium and calcium ion flow through specific ion channels. We found that targeting *CYP2J2* affected the expression of multiple sodium, potassium and calcium ion channels, several of which have been associated with the pathogenesis of cardiac dysrhythmias. One potential mechanism for this observation may be through *CYP2J2*’s regulation of EET bioactivation. Previous studies have demonstrated that EETs may act directly on ion channels, specifically L-type calcium channels and various potassium channels such as the K_ATP_ channel^14,15^. However, our interaction network analysis suggests that *CYP2J2* may also regulate other channel-associated proteins such as NOS2, HSPA5, RHOA, and CAMKK2 either through the formation of EETs or other signaling molecules yet to be determined.

Two other differentially up-regulated biological modules following CYP2J2 silencing were “Development” and “Extracellular Matrix”. Alteration of programs involved in heart structure and remodeling represents a critical mechanism leading to many types of cardiac disorders such as cardiomyopathies, dysrhythmias, and congenital heart disease. Our gene set enrichment analysis identified multiple such pathways including “heart valve formation”, “Glycosaminoglycan binding”, “Integrin-1 pathway”, “Syndecan-1 pathway”, “Embryonic morphogenesis”, and “Endocardial cushion development”. Furthermore, when we performed upstream regulator and causal network analysis to identify the drivers of cardiomyocyte response to *CYP2J2* silencing, we identified several well-known orchestrators of tissue remodeling and development such as HIF1-α, SHH, TGF-β, EDN1, and CTNNB1 (β-Catenin). These master regulators have been implicated in cardiac dysfunction, development, and repair^36–40^. Taken together, our findings highlight the widespread transcriptional landscape regulated by *CYP2J2* in human heart.

Despite its multifaceted role, it is important to note that when *CYP2J2* is knocked down, we do not observe increased cell death unless cardiomyocytes are exposed to external stressors such as reactive oxygen species, drug treatment or hypoxia^20^. It is therefore likely that the deleterious functional consequences of *CYP2J2* suppression in cardiac cells require a second pathophysiological “hit” to manifest its effect.

Our study has several limitations. Most importantly, we used an *in vitro* model to study the transcriptional consequences of silencing *CYP2J2*. While we utilized a well-characterized adult human cardiomyocyte system, extrapolating our findings to the intact heart remains to be demonstrated. While the main known function of *CYP2J2* is to oxidize AA to EETs, there may likely be other unknown endogenous substrates that mediate the widespread effects we observed in the transcriptome of cardiac cells. Identifying and functionally assessing the role of such mediators is beyond the scope of this paper and represents a future goal. Potential off-target effects of gene silencing are also a concern. The siRNAs used in these studies were validated to specifically knock down the target gene, however, there is a remote possibility that they may interact with other genes with downstream effects. Currently, the standard for ensuring specific target effects is to BLAST (https://blast.ncbi.nlm.nih.gov/Blast.cgi) the siRNA sequences to ensure specific alignment, all of which have been performed by Origene as part of their internal validation and quality control (Origene, Rockville, MD). Furthermore, we demonstrated that silencing of *CYP2J2* correlates with reduced enzymatic activity which is a result of reduced CYP2J2 expression. Changes in gene expression do not always correlate well with protein levels, and our reliance on transcriptomics in this study may not translate into functional effects in cardiomyocytes. However, the unbiased and comprehensive nature of RNA-seq provided a unique overview of cardiomyocyte programs regulated by CYP2J2 and allowed us to apply multi-level bioinformatics analyses that yielded several novel findings.

In summary, our study demonstrates a central role for *CYP2J2* in maintaining human cardiomyocyte homeostasis by regulating diverse transcriptional programs, particularly those involved in rhythmogenesis and tissue remodeling. These altered pathways and their drivers can potentially serve as future druggable targets to restore health in cardiovascular diseases.

## Methods

### Adult cardiac ventricular tissue

Discarded cardiac tissue from nine individuals (N = 9: 8 males, 1 female) with non-ischemic cardiomyopathy undergoing LVAD or transplant procedures were obtained from the University of Washington Medical Center for use in this study. The Institutional Review Board at University of Washington determined that since the tissue source was anonymous, it was not human research (NHR) and therefore waived the need for ethical review and informed consent. This policy is in accordance with Office for Human Research Protections guidelines (www.hhs.gov/ohrp/policy/cdebiol.html). Ventricular tissue was immediately flash-frozen in liquid nitrogen upon receipt and stored at −80 °C until thawed for processing. Upon thawing, tissue was washed with phosphate buffered saline and immediately processed.

Ventricular tissue from seventeen individuals (N = 17: 10 males, 7 females) with no known cardiac or cardiovascular health issues were used as the control group. These tissues were obtained from BioIVT (Westbury, NY). The samples were shipped under dry ice and upon receipt, were stored at −80 °C until further processing.

### Cardiac tissue processing

Proteomic analyses on cardiac tissues were initiated by homogenizing the tissues following the protocol by Aliwarga *et al*.^41^. Briefly, heart tissues were homogenized in an ice-cold 1x DPBS using Precellys24 (Bertin Instruments, Rockville, MD) at 6,800 rpm for 6 ×30 s with 60 s delay between cycles. In order to prevent further proteolysis, 1 × EDTA-free Halt cocktail protease inhibitor was added promptly to the heart homogenates. Total protein content of heart homogenates was determined using BCA^42^. After dilution of heart homogenates to 2 mg/mL total protein content, solubilization, enrichment, and extraction of membrane-bound proteins were performed using Thermo Fisher Mem-PER Plus membrane protein extraction kit (Thermo Fisher Scientific, Waltham, MA). Further sample preparation followed the published protocol by Xu *et al*.^43^. Briefly, 0.7 mg/mL human serum albumin and 2.7 μg/mL bovine serum albumin were added into membrane-bound or cytosolic fractions followed by protein denaturation and reduction step by adding 20 mM ammonium bicarbonate buffer, pH 7.8 and 17 mM dithiotrieitol at 95 °C for 10 minutes with gentle shaking. Once the denatured protein mixtures reached room temperature, they were alkylated by adding 59 mM of iodoacetamide and incubating the samples at room temperature in the dark for 30 min. The proteins were then extracted and pelleted using ice cold 5:1:4 of methanol:chloroform:water. After mixing and centrifugation, the upper and lower layers were removed, and the remaining pellets were dried at room temperature for 10 min. The pellets were washed with ice cold methanol followed by centrifugation and drying at room temperature for 30 min. To initiate trypsin digestion, 0.04 μg of trypsin was added into each pellet that was resuspended in 50 mM ammonium bicarbonate, pH 7.8, and incubated at 37 °C for 18 hours. The reaction was terminated by flash freezing the samples in dry ice. Finally, a cold cocktail of stable labelled peptide internal standard and 80% acetonitrile containing 0.5% formic acid were added to the samples. After mixing and centrifuging, the supernatants were collected and analyzed by mass spectrometry.

### Mass spectrometric assay for protein quantification

Quantification of CYP2J2 in the cardiac tissues were carried out using a validated mass spectrometric proteomics method as published in Xu *et al*.^43^. A stable labelled CYP2J2 peptide with [^13^C_6_, ^15^N_4_]- arginine or [^13^C_6_, ^15^N_2_]- lysine residues on the C-terminus and at least one surrogate peptide were used as an internal standard and for protein quantitation, respectively. The peptides used for the protein quantitation were VIGQGQQPSTAAR, EVTVDTTLAGYHLPK, and LLDEVTYLEASK. The mass spectrometry parameters for both the light and heavy peptides used are provided in Table 1. Detailed mass spectrometric assay was described by Xu *et al*.^43^. Briefly, samples were analyzed on an AB SCIEX Triple Quadrupole 6500 (PE SCIEX, Concord, ON, Canada) with a Waters Acquity UPLC, I-class (Waters Technologies, Milford, MA) interface in ESI positive ionization mode. The chromatographic separation of the peptides was achieved using a Waters Acquity UPLC HSS T3, 1.8 μm, C18, 100 Å, 2.1 ×100 mm column attached to a Phenomenex C18, 2 ×4 mm guard column. The mobile phase for this assay consisted of water containing 0.1% acetic acid and acetonitrile containing 0.1% acetic acid.

**Table 1:** Mass spectrometric parameters for peptides used in CYP2J2 quantification in human cardiac tissue.

Protein	Peptide sequence	Light/Heavy	Parent ion	Product ion	Retention time (min)	CE (eV)	DP (V)
**CYP2J2**	VIGQGQQPSTAAR	Light	656.85	915.46	8.3	32.5	69
				730.38			
				602.33			
		Heavy	661.86	925.47			
				740.39			
				612.33			81.1
	EVTVDTTLAGYHLPK	Light	548.63	785.43	14.2	22.4	61.1
				714.39			
				608.32			
		Heavy	551.3	793.44			
				722.41			
				612.33			71.5
	LLDEVTYLEASK	Light	690.87	910.49	14.8	33.7	71.5
				811.42			
				710.37			
		Heavy	694.87	918.5			
				819.43			
				718.39			70.9

### Adult cardiomyocyte culture protocol and *CYP2J2 and* scramble siRNA experiments

Cell culture materials including adult derived primary human cardiomyocytes (cat #36044-15), media (complete growth media, cat #M36044-15S), and extracellular matrix pre-coated flasks and plates (cat #E36044-15) were obtained from Celprogen Inc. (San Pedro, CA, USA). Media were further sterile filtered using a vacuum filter through a 0.22 μm polyethersulfone (PES) filter. Culturing and passage of cells followed previously published studies^6,20^.

*CYP2J2* gene expression was silenced following previously published protocols^20^ using the RNAiMAX lipofectamine (Thermo Fisher Scientific, Waltham MA) and the CYP2J2 Trilencer siRNA or scrambled siRNA (Origene, Rockville, MD), closely following the manufacturer’s suggested protocols. *HPRT1* silencing using Origene siRNA served as positive control. Lipofectamine was delivered using a reverse transfection protocol. siRNA was reconstituted to a stock concentration of 20 μM per the manufacturer protocols and prepared with the lipofectamine using OptiMEM reduced serum media (Thermo Fisher Scientific, Waltham, MA) by diluting to a concentration of 50 nM. The lipofectamine/siRNA solution was incubated at room temperature for at least 10 minutes, during which time cells were washed, trypsinized, pelleted, resuspended and diluted to a concentration of 200,000 cells/mL in complete media. The lipofectamine/siRNA stocks were added to each well of a 12-well plate to a final concentration of 10 nM siRNA (250 μL volume of 50 nM siRNA/lipofectamine in OptiMEM), followed by the cells (1 mL of cell suspension) for a final volume of 1250 μL in each well. The cells were incubated with the lipofectamine/siRNA for 72 hours. Following gene silencing, cells were harvested and stored until total RNA isolation.

We performed two independent sets of experiments on human cardiomyoctye cultures treated with *CYP2J2* siRNA (n = 3) or scrambled siRNA (n = 3). The first set of studies was used for genome-wide RNA-seq analysis (n = 6 total), and the second set of experiments was performed for qPCR validation purposes (n = 6 total).

### Terfenadine activity assay

CYP2J2 enzymatic activity following gene silencing was determined using previously published protocols^6^. Briefly, cells were treated with lipofectamine and either scramble or CYP2J2 siRNA as described above for 72 hours. After 72 hours, the cells were washed and treated with serum free media containing 1.5 μM terfenadine, an established CYP2J2 substrate. Mass spectrometry was used to determine relative levels of hydroxyterfenadine using previously published parameters^6^, using midazolam (0.05 nM final concentration) as an internal standard for relative quantification and 10 mM ammonium acetate in water and acetonitrile as the mobile phase (aqueous and organic, respectively).

### Total RNA isolation and qPCR

Total RNA was extracted using the MagMax 96 Total RNA Isolation kit (Thermo Fisher Scientific, Waltham MA). Initial RNA quality (A260/A280) and quantity was determined using a Synergy HTX Multi-Mode Reader (BioTek, Winooski VT). In order to check the CYP2J2 siRNA knockdown efficiency, total RNA was used to synthesize cDNA using the High Capacity RNA-to-cDNA kit (Thermo Fisher Scientific, Waltham MA). RT-PCR was then carried out using TaqMan (Thermo Fisher Scientific, Waltham MA) FAM reporter primers for *CYP2J2* as well as a housekeeping gene, *GUSB*. Additionally, a select subset of genes were quantified using RT-PCR to validate RNA-seq findings in a separate experiment. Specifically, we used TaqMan primers for *HMOX1, SCN1B, PSAT1, KCNA2*, and *GLTP* to assay for the expression levels of these genes by RT-PCR following *CYP2J2* knockdown. Cycle threshold (C_T_) values and the ΔC_T_ method followed by the 2^ΔCT^ calculation were used to determine the relative quantity of CYP2J2 (and other genes) mRNA present relative to the *GUSB*. The mRNA levels were first normalized to the housekeeping gene using the ΔC_T_ method and then the levels of expression in treated cells were compared to expression levels in untreated cells using the ΔΔC_T_ calculation and relative gene expression levels were reported using the 2^−ΔΔCT^ calculation^44^.

### RNA-sequencing

Total RNA from adult cardiomyocytes treated with *CYP2J2* siRNA or scrambled siRNA (n = 3 per group) was isolated using the methods described above. RNA-seq was performed at University of Washington’s Northwest Genomics Center (http://nwgc.gs.washington.edu/). Briefly, library generation was performed using TruSeq Stranded mRNA library prep kit (Illumina, San Diego, CA) using 1 μg total RNA. Sequencing was performed using an Illumina HiSeq 4000 instrument using paired end 75 bp lanes. Base calls were generated in real-time on the HiSeq instrument, and RTA-generated BCL files were converted to FASTQ files. Sequence read and base quality were checked using the FASTX-toolkit (http://hannonlab.cshl.edu/fastx_toolkit/) and FastQC (http://www.bioinformatics.babraham.ac.uk/projects/fastqc/), and adaptor and low-quality bases were trimmed. Sequences were aligned to GRCh38 with reference transcriptome Gencode release 26 (GRCh38.p10) using STAR (v2.5.3a). Transcript abundances were quantified using Kallisto (v0.43.1)^45^. All RNA-seq data meeting MINSEQE (Minimum Information About a Next-generation Sequencing Experiment) have been deposited at Gene Expression Omnibus repository (https://www.ncbi.nlm.nih.gov/geo/, GSE136957).

### Gene expression analysis

Initially, we applied correspondence analysis^46^, a method for reducing high dimensional data, to all measured transcripts across experimental conditions after regularized log transformation of the raw counts^47^. Next, we identified differentially expressed genes between *CYP2J2* knockdown vs. scrambled siRNA cardiomyocytes using DESeq2^47^ in the R statistical environment. Multiple hypothesis testing was addressed using Benjamini-Hochberg’s false discovery rate (FDR) analysis with an FDR < 0.01 designating significant differential gene expression^47^.

### Bioinformatics and pathway analyses

To identify transcriptional programs altered by selective siRNA knockdown of *CYP2J2* in human cardiomyocytes, we applied Gene Set Enrichment Analysis (GSEA)^48^ using approximately 7,000 well-curated gene sets comprised of canonical biological pathways (e.g., Hallmark, KEGG, Reactome, Biocarta) and Gene Ontology (GO) annotations to all identified unique transcripts rank ordered based on DESeq2’s test statistic. An FDR < 0.05 threshold for significant enrichment was used from possibly anti-conservative *P*-values computed based on 1000 random gene set selections^48^. Network visualization of GSEA results was performed using Enrichment Map^49^, a plug-in application within the bioinformatics software platform, Cytoscape^50^. To generate the pathway enrichment network, all identified significant gene sets (FDR < 0.05) were used as nodes. Edges were drawn between pathways if at least 50% of their gene members overlapped. Distinct biological modules emerged based on the network topology and consisted of aggregates of highly connected gene sets. We performed leading edge analysis^51^ on the gene sets that mapped to the “Ion Channel Signaling” GSEA module in order to identify transcripts with the largest contribution to pathway enrichment, and further refined these genes to those differentially expressed at FDR < 0.01. We next performed gene product interaction analysis using Ingenuity (IPA, Qiagen Bioinformatics, Redwood City, CA) on these “Ion Channel Signaling” associated genes, limiting relationships among nodes to those based on high confidence direct interactions. Upstream regulator and causal network analysis (IPA) was performed on all differentially expressed genes (FDR < 0.01). In this approach, critical orchestrators of the transcriptional response are identified by comparing the direction of change observed among differentially expressed genes following *CYP2J2* silencing and expected effects based on prior knowledge (i.e., published literature, canonical pathways)^52,53^.

## Supplementary information

Supplementary table S1

Supplementary table S2

Supplementary table S3

Supplementary table S4

Supplementary table S5

## Data Availability

All RNA-seq data meeting MINSEQE (Minimum Information About a Next-generation Sequencing Experiment) have been deposited at Gene Expression Omnibus repository (https://www.ncbi.nlm.nih.gov/geo/, GSE136957). Additionally, all RNA-seq data collected in this study are available upon reasonable request from the corresponding author.
