# Supplementary material for: CYP2J2 Modulates Diverse Transcriptional Programs in Adult Human Cardiomyocytes
Source: Sci Rep. 2020 Mar 24;10:5329. doi: 10.1038/s41598-020-62174-w (PMC7093536; doi:10.1038/s41598-020-62174-w)
Supplement: Supplementary file 1 — Supplementary table S1 [file 41598_2020_62174_MOESM1_ESM.pdf]

Table S1. List of top 25 differentially up- and down-regulated genes in CYP2J2-silenced cardiomyoctyes.

Up-regulated				
**Gene Symbol**	**Gene Name**	**log_2[FoldChange]**	**P-value**	**FDR**
PSAT1	phosphoserine aminotransferase 1	1.828	3.67E-77	6.96E-73
PGAM1	phosphoglycerate mutase 1	1.575	4.62E-67	4.37E-63
CBX1	chromobox 1	1.157	6.65E-65	4.20E-61
SATB2	SATB homeobox 2	0.942	1.54E-60	7.27E-57
SHISA2	shisa family member 2	2.320	4.27E-60	1.62E-56
ZDHHC9	zinc finger DHHC-type containing 9	0.941	1.32E-59	4.17E-56
LIX1L	limb and CNS expressed 1 like	1.150	8.76E-58	2.37E-54
FUBP3	far upstream element binding protein 3	0.814	1.32E-49	3.13E-46
UBE3C	ubiquitin protein ligase E3C	0.953	1.23E-47	2.60E-44
HMOX1	heme oxygenase 1	1.828	7.48E-45	1.42E-41
MAPRE1	microtubule associated protein RP/EB family member 1	1.131	1.81E-42	3.12E-39
ST13	suppression of tumorigenicity 13	0.696	3.67E-40	5.79E-37
SLC13A4	solute carrier family 13 member 4	1.266	3.62E-38	5.27E-35
ENOPH1	enolase-phosphatase 1	0.957	1.61E-33	2.17E-30
ADCYAP1R1	ADCYAP receptor type I	1.948	1.53E-27	1.38E-24
DNAJB6	DnaJ heat shock protein family	0.605	2.75E-27	2.37E-24
EZR	ezrin	0.955	2.31E-24	1.83E-21
KCNA2	potassium voltage-gated channel subfamily A member 2	1.939	2.83E-24	2.10E-21
COL14A1	collagen type XIV alpha 1 chain	0.959	1.69E-23	1.19E-20
TNFRSF10B	TNF receptor superfamily member 10b	0.516	2.43E-23	1.64E-20
CRIM1	cysteine rich transmembrane BMP regulator 1	0.780	2.19E-22	1.43E-19
SYT2	synaptotagmin 2	1.254	4.69E-22	2.77E-19
MAF	MAF bZIP transcription factor	0.797	5.46E-21	3.04E-18
ABAT	4-aminobutyrate aminotransferase	0.766	6.84E-21	3.70E-18
IGF2BP1	insulin like growth factor 2 mRNA binding protein 1	0.408	8.42E-21	4.31E-18

Down-regulated				
**Gene Symbol**	**Gene Name**	**log_2[FoldChange]**	**P-value**	**FDR**
NEK9	NIMA related kinase 9	-0.492	1.31E-29	1.66E-26
GLTP	glycolipid transfer protein	-1.109	1.89E-29	2.24E-26
ZCCHC14	zinc finger CCHC-type containing 14	-0.576	2.76E-29	3.07E-26
DNAJC1	DnaJ heat shock protein family	-0.972	1.43E-28	1.50E-25
ARPC2	actin related protein 2/3 complex subunit 2	-0.797	6.28E-28	6.26E-25
PTPN21	protein tyrosine phosphatase, non-receptor type 21	-0.746	1.31E-27	1.24E-24
HMGN2	high mobility group nucleosomal binding domain 2	-0.625	1.28E-24	1.06E-21
HOXA10	homeobox A10	-0.557	2.89E-24	2.10E-21
CORO1C	coronin 1C	-0.685	2.49E-22	1.57E-19
ANXA1	annexin A1	-0.917	3.07E-22	1.87E-19
SIK2	salt inducible kinase 2	-0.536	2.96E-21	1.70E-18
RBM47	RNA binding motif protein 47	-0.831	7.83E-21	4.12E-18
ARL4C	ADP ribosylation factor like GTPase 4C	-0.790	2.59E-20	1.26E-17
DPY30	dpy-30, histone methyltransferase complex regulatory subunit	-0.861	4.00E-20	1.90E-17
ULK2	unc-51 like autophagy activating kinase 2	-0.649	4.87E-20	2.25E-17
DUSP16	dual specificity phosphatase 16	-0.684	1.82E-18	7.33E-16
STX3	syntaxin 3	-0.639	5.88E-18	2.23E-15
TGFBR2	transforming growth factor beta receptor 2	-0.874	9.71E-18	3.61E-15
SMIM10L1	small integral membrane protein 10 like 1	-0.754	4.70E-17	1.65E-14
TFDP1	transcription factor Dp-1	-0.414	9.21E-17	3.01E-14
BSPRY	B-box and SPRY domain containing	-1.529	1.41E-16	4.45E-14
CYP2J2	cytochrome P450 family 2 subfamily J member 2	-1.084	1.82E-16	5.67E-14
ACLY	ATP citrate lyase	-0.811	2.11E-16	6.45E-14
GTF2H1	general transcription factor IIH subunit 1	-0.907	1.40E-15	4.04E-13
SSH1	slingshot protein phosphatase 1	-0.520	1.41E-15	4.04E-13
