# Supplementary material for: CYP2J2 Modulates Diverse Transcriptional Programs in Adult Human Cardiomyocytes
Source: Sci Rep. 2020 Mar 24;10:5329. doi: 10.1038/s41598-020-62174-w (PMC7093536; doi:10.1038/s41598-020-62174-w)
Supplement: Supplementary file 2 — Supplementary table S2 [file 41598_2020_62174_MOESM2_ESM.pdf]

Table S2. List of differentially up-regulated genes sets in CYP2J2-silenced cardiomyoctes (FDR < 0.05).

Gene Set Name	Number of members	FDR
GO_CATION_CHANNEL_COMPLEX	100	0
GO_PROTEINACEOUS_EXTRACELLULAR_MATRIX	241	0
GO_EXTRACELLULAR_MATRIX	286	0
GO_COLLAGEN_BINDING	48	0
GO_ENDOCARDIAL_CUSHION_DEVELOPMENT	30	0
GO_ENDOCARDIAL_CUSHION_MORPHOGENESIS	22	0
GO_CALCIUM_ION_BINDING	439	0
GO_CELL_FATE_COMMITMENT	173	0
NABA_CORE_MATRISOME	182	0
GO_EXOCYTIC_VESICLE_MEMBRANE	44	0
GO_VOLTAGE_GATED_CATION_CHANNEL_ACTIVITY	75	0
KEGG_NEUROACTIVE_LIGAND_RECEPTOR_INTERACTION	124	0
GO_TRANSPORTER_COMPLEX	190	7.01E-05
GO_MULTICELLULAR_ORGANISMAL_SIGNALING	83	7.51E-05
GO_VOLTAGE_GATED_ION_CHANNEL_ACTIVITY	113	8.09E-05
GO_POSITIVE_REGULATION_OF_KIDNEY_DEVELOPMENT	32	1.24E-04
GO_NEURON_FATE_COMMITMENT	54	1.32E-04
GO_CELL_FATE_SPECIFICATION	53	4.13E-04
NABA_MATRISOME_ASSOCIATED	370	5.03E-04
GO_VOLTAGE_GATED_POTASSIUM_CHANNEL_ACTIVITY	49	6.37E-04
GO_MESONEPHRIC_TUBULE_MORPHOGENESIS	46	6.76E-04
GO_TRANSMISSION_OF_NERVE_IMPULSE	33	7.08E-04
GO_REGULATION_OF_MORPHOGENESIS_OF_A_BRANCHING_STRUCTURE	41	8.42E-04
REACTOME_POTASSIUM_CHANNELS	61	8.79E-04
GO_POTASSIUM_CHANNEL_ACTIVITY	67	9.01E-04
GO_BRANCHING_INVOLVED_IN_URETERIC_BUD_MORPHOGENESIS	39	9.37E-04
GO_EXTRACELLULAR_STRUCTURE_ORGANIZATION	229	9.86E-04
GO_GATED_CHANNEL_ACTIVITY	188	1.22E-03
NABA_ECM_GLYCOPROTEINS	131	1.35E-03
GO_EXTRACELLULAR_MATRIX_STRUCTURAL_CONSTITUENT	50	1.36E-03
GO_EMBRYONIC_SKELETAL_SYSTEM_DEVELOPMENT	108	1.37E-03
REACTOME_GPCR_LIGAND_BINDING	165	1.38E-03
GO_ENDOCARDIAL_CUSHION_FORMATION	15	1.41E-03
HALLMARK_KRAS_SIGNALING_DN	116	1.45E-03
GO_POSITIVE_REGULATION_OF_VACUOLAR_TRANSPORT	14	1.50E-03
NABA_SECRETED_FACTORS	158	1.86E-03
GO_MYELIN_SHEATH	150	1.99E-03
GO_POTASSIUM_CHANNEL_COMPLEX	55	2.03E-03
GO_VOCALIZATION_BEHAVIOR	11	2.05E-03
GO_EPITHELIAL_TO_MESENCHYMAL_TRANSITION	50	2.11E-03
KEGG_ANTIGEN_PROCESSING_AND_PRESENTATION	46	2.11E-03
GO_DELAYED_RECTIFIER_POTASSIUM_CHANNEL_ACTIVITY	21	2.11E-03
GO_GLYCOSAMINOGLYCAN_BINDING	113	2.13E-03
REACTOME_VOLTAGE_GATED_POTASSIUM_CHANNELS	26	2.48E-03

GO_ANATOMICAL_STRUCTURE_MATURATION	31	2.49E-03
GO_MESONEPHROS_DEVELOPMENT	79	2.50E-03
GO_MESENCHYME_MORPHOGENESIS	37	2.53E-03
GO_REGULATION_OF_VACUOLAR_TRANSPORT	28	2.56E-03
GO_MAIN_AXON	42	2.56E-03
GO_G_PROTEIN_COUPLED_RECEPTOR_ACTIVITY	189	2.57E-03
GO_SKELETAL_SYSTEM_DEVELOPMENT	371	2.61E-03
GO_METALLOENDOPEPTIDASE_ACTIVITY	80	2.73E-03
GO_CARDIAC_CONDUCTION	58	2.84E-03
GO_COLLAGEN_TRIMER	54	2.86E-03
KEGG_ECM_RECEPTOR_INTERACTION	65	3.01E-03
NABA_ECM_REGULATORS	133	3.01E-03
GO_RESPONSE_TO_BMP	73	3.42E-03
GO_REGULATION_OF_POSTSYNAPTIC_MEMBRANE_POTENTIAL	37	4.13E-03
GO_REGULATION_OF_AMINE_TRANSPORT	39	4.16E-03
GO_PEPTIDYL_CYSTEINE_MODIFICATION	17	4.16E-03
GO_T_TUBULE	37	4.18E-03
GO_REGULATION_OF_CHONDROCYTE_DIFFERENTIATION	38	4.19E-03
GO_KIDNEY_MORPHOGENESIS	67	4.21E-03
GO_SARCOLEMMA	100	4.28E-03
GO_BRANCHING_MORPHOGENESIS_OF_AN_EPITHELIAL_TUBE	118	5.06E-03
REACTOME_EXTRACELLULAR_MATRIX_ORGANIZATION	63	5.07E-03
GO_CARDIAC_EPITHELIAL_TO_MESENCHYMAL_TRANSITION	22	5.11E-03
GO_CALCIUM_ION_REGULATED_EXOCYTOSIS_OF_NEUROTRANSMITTER	23	5.15E-03
GO_FEMALE_SEX_DIFFERENTIATION	90	5.26E-03
GO_SMOOTH_MUSCLE_CONTRACTION	33	5.30E-03
GO_GLIAL_CELL_FATE_COMMITMENT	10	5.55E-03
GO_HYALURONIC_ACID_BINDING	15	5.57E-03
GO_SPINAL_CORD_DEVELOPMENT	85	5.59E-03
GO_NEGATIVE_REGULATION_OF_CARTILAGE_DEVELOPMENT	18	5.74E-03
GO_REGULATION_OF_MESONEPHROS_DEVELOPMENT	20	6.15E-03
GO_POSITIVE_REGULATION_OF_TRANSMEMBRANE_RECEPTOR_PROTEIN_[illegible]	81	6.15E-03
GO_OSSIFICATION	205	6.19E-03
GO_EMBRYONIC_ORGAN_MORPHOGENESIS	238	6.23E-03
GO_HEART_VALVE_FORMATION	10	6.25E-03
HALLMARK_HEDGEHOG_SIGNALING	33	6.29E-03
GO_EXOCYTIC_VESICLE	115	6.32E-03
GO_EAR_DEVELOPMENT	156	6.32E-03
GO_HOMOPHILIC_CELL_ADHESION_VIA_PLASMA_MEMBRANE_ADHESION_[illegible]	72	6.33E-03
GO_MORPHOGENESIS_OF_A_BRANCHING_STRUCTURE	143	6.36E-03
GO_EXTRACELLULAR_MATRIX_BINDING	39	6.43E-03
GO_NEURON_PROJECTION_GUIDANCE	176	6.46E-03
GO_POST_EMBRYONIC_MORPHOGENESIS	11	7.17E-03
GO_POTASSIUM_ION_TRANSPORT	91	7.21E-03
GO_RENAL_TUBULE_DEVELOPMENT	65	7.70E-03
GO_NODE_OF_RANVIER	11	7.74E-03
GO_SYNAPSE_PART	463	7.82E-03

GO_MHC_CLASS_II_PROTEIN_COMPLEX_BINDING	12	7.91E-03
GO_METAL_ION_TRANSMEMBRANE_TRANSPORTER_ACTIVITY	263	7.99E-03
GO_POSITIVE_REGULATION_OF_AMINE_TRANSPORT	19	8.07E-03
GO_REGULATION_OF_TRANSMEMBRANE_RECEPTOR_PROTEIN_SERINE_TH	164	8.31E-03
GO_CATION_CHANNEL_ACTIVITY	172	9.16E-03
GO_SKELETAL_SYSTEM_MORPHOGENESIS	170	9.94E-03
GO_RETINA_MORPHOGENESIS_IN_CAMERA_TYPE_EYE	32	9.99E-03
GO_SYNAPTIC_SIGNALING	268	1.00E-02
REACTOME_G_ALPHA_S_SIGNALLING_EVENTS	62	1.01E-02
GO_METANEPHROS_DEVELOPMENT	65	1.01E-02
GO_POSITIVE_REGULATION_OF_ION_TRANSPORT	158	1.01E-02
GO_REGULATION_OF_KIDNEY_DEVELOPMENT	44	1.01E-02
GO_NEPHRON_EPITHELIUM_DEVELOPMENT	77	1.01E-02
GO_CELL_CELL_ADHESION_VIA_PLASMA_MEMBRANE_ADHESION_MOLECU	94	1.04E-02
GO_SODIUM_ION_TRANSMEMBRANE_TRANSPORT	56	1.07E-02
GO_RESPONSE_TO_IRON_ION	30	1.09E-02
REACTOME_COLLAGEN_FORMATION	48	1.09E-02
PID_INTEGRIN1_PATHWAY	56	1.12E-02
GO_SENSORY_ORGAN_MORPHOGENESIS	187	1.13E-02
GO_LOCOMOTORY_BEHAVIOR	130	1.14E-02
GO_MHC_PROTEIN_COMPLEX_BINDING	12	1.14E-02
KEGG_GLYCOLYSIS_GLUCONEOGENESIS	41	1.15E-02
GO_CELL_MORPHOGENESIS_INVOLVED_IN_DIFFERENTIATION	421	1.16E-02
GO_SEMAPHORIN_RECEPTOR_COMPLEX	11	1.16E-02
GO_POSITIVE_REGULATION_OF_MESONEPHROS_DEVELOPMENT	18	1.18E-02
GO_MUSCLE_SYSTEM_PROCESS	204	1.18E-02
GO_REGULATION_OF_BLOOD_PRESSURE	105	1.19E-02
GO_FEMALE_GENITALIA_DEVELOPMENT	11	1.19E-02
GO_REGULATION_OF_NEUROTRANSMITTER_LEVELS	138	1.20E-02
GO_DORSAL_SPINAL_CORD_DEVELOPMENT	13	1.21E-02
GO_MEMBRANE_DEPOLARIZATION_DURING_CARDIAC_MUSCLE_CELL_ACTI	12	1.22E-02
GO_NEGATIVE_REGULATION_OF_PLATELET_ACTIVATION	15	1.23E-02
GO_EXTRACELLULAR_MATRIX_COMPONENT	99	1.26E-02
GO_REGULATION_OF_EARLY_ENDOSOME_TO_LATE_ENDOSOME_TRANSPO	16	1.31E-02
GO_REGULATION_OF_HEART_CONTRACTION	151	1.31E-02
GO_CELL_DIFFERENTIATION_IN_SPINAL_CORD	42	1.33E-02
GO_REGULATION_OF_CARTILAGE_DEVELOPMENT	51	1.40E-02
GO_FOREBRAIN_REGIONALIZATION	20	1.45E-02
GO_MESENCHYME_DEVELOPMENT	153	1.45E-02
GO_GLYCOPROTEIN_COMPLEX	14	1.46E-02
REACTOME_AMINE_LIGAND_BINDING_RECEPTORS	15	1.47E-02
GO_POST_ANAL_TAIL_MORPHOGENESIS	14	1.47E-02
GO_PEPTIDE_RECEPTOR_ACTIVITY	56	1.47E-02
GO_REGULATION_OF_BLOOD_CIRCULATION	198	1.47E-02
GO_METANEPHRIC_NEPHRON_MORPHOGENESIS	17	1.48E-02
GO_DORSAL_VENTRAL_PATTERN_FORMATION	74	1.49E-02
GO_ORGAN_MATURATION	15	1.49E-02

GO_SULFATION	11	1.50E-02
GO_KIDNEY_EPITHELIUM_DEVELOPMENT	108	1.50E-02
REACTOME_CLASS_B_2_SECRETIN_FAMILY_RECEPTORS	55	1.50E-02
GO_PASSIVE_TRANSMEMBRANE_TRANSPORTER_ACTIVITY	271	1.50E-02
GO_REGULATION_OF_SYSTEM_PROCESS	337	1.52E-02
GO_ANATOMICAL_STRUCTURE_ARRANGEMENT	16	1.52E-02
GO_TRANSFERASE_ACTIVITY_TRANSFERRING_NITROGENOUS_GROUPS	14	1.53E-02
GO_HEPARIN_BINDING	87	1.53E-02
REACTOME_GPCR_DOWNSTREAM_SIGNALING	251	1.53E-02
GO_OSTEOBLAST_DIFFERENTIATION	109	1.56E-02
HALLMARK_EPITHELIAL_MESENCHYMAL_TRANSITION	159	1.56E-02
GO_REGULATION_OF_CATECHOLAMINE_SECRETION	22	1.56E-02
GO_AXON	323	1.57E-02
GO_REGIONALIZATION	257	1.57E-02
GO_VOLTAGE_GATED_CALCIUM_CHANNEL_COMPLEX	23	1.61E-02
GO_EMBRYONIC_SKELETAL_SYSTEM_MORPHOGENESIS	83	1.61E-02
NABA_COLLAGENS	38	1.62E-02
GO_HEART_VALVE_DEVELOPMENT	28	1.63E-02
GO_METANEPHRIC_NEPHRON_DEVELOPMENT	22	1.65E-02
GO_METANEPHRIC_RENAL_VESICLE_MORPHOGENESIS	9	1.65E-02
GO_MEMBRANE_DEPOLARIZATION_DURING_ACTION_POTENTIAL	26	1.66E-02
GO_G_PROTEIN_COUPLED_RECEPTOR_SIGNALING_PATHWAY	359	1.66E-02
GO_PRESYNAPSE	214	1.66E-02
GO_PRESYNAPTIC_MEMBRANE	40	1.66E-02
GO_SYNAPTIC_MEMBRANE	185	1.67E-02
GO_LAMININ_BINDING	26	1.67E-02
GO_VENTRAL_SPINAL_CORD_DEVELOPMENT	38	1.77E-02
KEGG_GLYCINE_SERINE_AND_THREONINE_METABOLISM	23	1.77E-02
GO_NEPHRON_DEVELOPMENT	95	1.80E-02
GO_CELL_CELL_SIGNALING	451	1.81E-02
GO_PYRUVATE_METABOLIC_PROCESS	48	1.83E-02
GO_CARTILAGE_DEVELOPMENT	122	1.83E-02
REACTOME_CLASS_A1_RHODOPSIN_LIKE_RECEPTORS	103	1.98E-02
NABA_PROTEOGLYCANS	13	1.98E-02
GO_REGULATION_OF_PATHWAY_RESTRICTED_SMAD_PROTEIN_PHOSPHOR	46	1.98E-02
GO_TAXIS	310	1.99E-02
GO_TELENCEPHALON_REGIONALIZATION	11	1.99E-02
GO_BONE_DEVELOPMENT	129	1.99E-02
KEGG_CALCIUM_SIGNALING_PATHWAY	125	1.99E-02
GO_MUSCLE_CONTRACTION	166	2.00E-02
GO_CALCIUM_CHANNEL_COMPLEX	38	2.05E-02
BIOCARTA_UCALPAIN_PATHWAY	17	2.06E-02
GO_CELL_FATE_DETERMINATION	33	2.06E-02
GO_NEURONAL_CELL_BODY_MEMBRANE	14	2.07E-02
GO_POTASSIUM_ION_HOMEOSTASIS	12	2.08E-02
GO_ADULT_BEHAVIOR	98	2.17E-02
REACTOME_SIGNALING_BY_GPCR	327	2.18E-02

GO_DIENCEPHALON_DEVELOPMENT	57	2.19E-02
GO_CARDIAC_MUSCLE_CELL_ACTION_POTENTIAL	28	2.23E-02
GO_MODULATION_OF_SYNAPTIC_TRANSMISSION	217	2.24E-02
GO_POSITIVE_REGULATION_OF_ASTROCYTE_DIFFERENTIATION	10	2.25E-02
GO_SODIUM_CHANNEL_COMPLEX	10	2.25E-02
GO_NEURON_FATE_SPECIFICATION	24	2.25E-02
GO_AUTONOMIC_NERVOUS_SYSTEM_DEVELOPMENT	32	2.26E-02
GO_POSITIVE_REGULATION_OF_PATHWAY_RESTRICTED_SMAD_PROTEIN_F	36	2.26E-02
REACTOME_PLATELET_HOMEOSTASIS	59	2.28E-02
GO_RENAL_VESICLE_DEVELOPMENT	12	2.33E-02
GO_SMOOTH_MUSCLE_CELL_DIFFERENTIATION	27	2.34E-02
GO_EAR_MORPHOGENESIS	94	2.35E-02
GO_VOLTAGE_GATED_SODIUM_CHANNEL_ACTIVITY	13	2.39E-02
GO_POSITIVE_REGULATION_OF_SKELETAL_MUSCLE_TISSUE_DEVELOPMEN	22	2.41E-02
GO_REGULATION_OF_CELL_PROJECTION_SIZE	8	2.43E-02
GO_REGULATION_OF_ION_TRANSPORT	386	2.46E-02
GO_NEUROTRANSMITTER_TRANSPORT	105	2.51E-02
GO_EMBRYONIC_ORGAN_DEVELOPMENT	345	2.54E-02
GO_AXON_PART	164	2.58E-02
GO_BEHAVIOR	369	2.58E-02
GO_VOLTAGE_GATED_SODIUM_CHANNEL_COMPLEX	9	2.59E-02
REACTOME_INTERACTION_BETWEEN_L1_AND_ANKYRINS	17	2.59E-02
GO_PRESYNAPTIC_PROCESS_INVOLVED_IN_SYNAPTIC_TRANSMISSION	83	2.59E-02
GO_NEPHRON_TUBULE_FORMATION	12	2.59E-02
GO_MHC_PROTEIN_COMPLEX	17	2.60E-02
KEGG_GRAFT_VERSUS_HOST_DISEASE	16	2.60E-02
GO_REGULATION_OF_HEART_RATE_BY_CARDIAC_CONDUCTION	23	2.60E-02
GO_CARDIAC_SEPTUM_MORPHOGENESIS	45	2.60E-02
REACTOME_GLUCAGON_TYPE_LIGAND_RECEPTORS	18	2.60E-02
GO_NERVE_DEVELOPMENT	57	2.60E-02
GO_CELLULAR_POTASSIUM_ION_HOMEOSTASIS	10	2.61E-02
GO_REPLACEMENT_OSSIFICATION	23	2.61E-02
GO_BONE_MORPHOGENESIS	67	2.61E-02
GO_OXIDOREDUCTASE_ACTIVITY_ACTING_ON_THE_CH_NH2_GROUP_OF_D	13	2.68E-02
GO_SEX_DIFFERENTIATION	199	2.69E-02
GO_SYNAPSE_ORGANIZATION	111	2.69E-02
GO_ADENYLATE_CYCLASE_MODULATING_G_PROTEIN_COUPLED_RECEPTO	76	2.71E-02
GO_REGULATION_OF_ENDOTHELIAL_CELL_DIFFERENTIATION	20	2.71E-02
GO_ACTION_POTENTIAL	63	2.71E-02
GO_SENSORY_PERCEPTION_OF_PAIN	46	2.74E-02
PID_SYNDECAN_1_PATHWAY	37	2.74E-02
GO_CELL_FATE_COMMITMENT_INVOLVED_IN_FORMATION_OF_PRIMARY_GI	21	2.74E-02
GO_PATTERN_SPECIFICATION_PROCESS	346	2.75E-02
GO_NEGATIVE_REGULATION_OF_CHONDROCYTE_DIFFERENTIATION	13	2.75E-02
GO_EYE_MORPHOGENESIS	103	2.76E-02
GO_EPITHELIAL_TO_MESENCHYMAL_TRANSITION_INVOLVED_IN_ENDOCAR	11	2.79E-02
REACTOME_CYTOSOLIC_SULFONATION_OF_SMALL_MOLECULES	9	2.81E-02

GO_PERIKARYON	67	2.81E-02
GO_G_PROTEIN_COUPLED_AMINE_RECEPTOR_ACTIVITY	20	2.82E-02
REACTOME_OTHER_SEMAPHORIN_INTERACTIONS	12	2.86E-02
GO_POSITIVE_REGULATION_OF_ENDOTHELIAL_CELL_DIFFERENTIATION	10	2.87E-02
GO_SINGLE_ORGANISM_BEHAVIOR	279	2.87E-02
GO_CELL_MORPHOGENESIS_INVOLVED_IN_NEURON_DIFFERENTIATION	299	2.87E-02
GO_SODIUM_ION_TRANSMEMBRANE_TRANSPORTER_ACTIVITY	78	2.91E-02
GO_NEGATIVE_REGULATION_OF_AMINE_TRANSPORT	16	2.93E-02
GO_SPHINGOLIPID_MEDIATED_SIGNALING_PATHWAY	9	2.98E-02
KEGG_DILATED_CARDIOMYOPATHY	67	3.04E-02
GO_NEGATIVE_REGULATION_OF_COAGULATION	29	3.12E-02
GO_POSITIVE_REGULATION_OF_NEURAL_PRECURSOR_CELL_PROLIFERATI	33	3.12E-02
GO_POSITIVE_REGULATION_OF_NERVOUS_SYSTEM_DEVELOPMENT	347	3.13E-02
GO_TRABECULA_FORMATION	18	3.13E-02
GO_LIPOPROTEIN_PARTICLE_RECEPTOR_ACTIVITY	12	3.14E-02
GO_ENDOCRINE_SYSTEM_DEVELOPMENT	98	3.14E-02
GO_CONNECTIVE_TISSUE_DEVELOPMENT	157	3.14E-02
GO_VOLTAGE_GATED_CALCIUM_CHANNEL_ACTIVITY	24	3.14E-02
GO_POSITIVE_REGULATION_OF_EPITHELIAL_CELL_PROLIFERATION	119	3.14E-02
GO_NEURAL_RETINA_DEVELOPMENT	36	3.15E-02
GO_SULFUR_COMPOUND_BINDING	152	3.18E-02
GO_MULTICELLULAR_ORGANISMAL_RESPONSE_TO_STRESS	44	3.23E-02
GO_GLUTAMATE_RECEPTOR_SIGNALING_PATHWAY	27	3.23E-02
GO_CAMERA_TYPE_EYE_MORPHOGENESIS	78	3.25E-02
GO_GROWTH_FACTOR_ACTIVITY	99	3.25E-02
GO_NEURON_PROJECTION_MORPHOGENESIS	328	3.26E-02
GO_REGULATION_OF_CALCIUM_ION_DEPENDENT_EXOCYTOSIS	62	3.26E-02
GO_CENTRAL_NERVOUS_SYSTEM_NEURON_DIFFERENTIATION	133	3.27E-02
GO_FORELIMB_MORPHOGENESIS	34	3.29E-02
GO_DEVELOPMENTAL_INDUCTION	23	3.31E-02
GO_CRANIAL_NERVE_DEVELOPMENT	35	3.34E-02
GO_MESENCHYMAL_CELL_DIFFERENTIATION	113	3.35E-02
GO_SEMAPHORIN_PLEXIN_SIGNALING_PATHWAY_INVOLVED_IN_NEURON_F	13	3.36E-02
GO_ATRIOVENTRICULAR_VALVE_DEVELOPMENT	17	3.36E-02
GO_POSITIVE_REGULATION_OF_METANEPHROS_DEVELOPMENT	10	3.36E-02
GO_POSITIVE_REGULATION_OF_SODIUM_ION_TRANSPORT	30	3.36E-02
GO_REGULATION_OF_DOPAMINE_SECRETION	12	3.36E-02
GO_EMBRYONIC_FORELIMB_MORPHOGENESIS	28	3.37E-02
GO_LYMPHOCYTE_MIGRATION	17	3.38E-02
GO_LUNG_ALVEOLUS_DEVELOPMENT	35	3.42E-02
GO_PLASMA_MEMBRANE_PROTEIN_COMPLEX	336	3.43E-02
GO_REGULATION_OF_TRANSMEMBRANE_TRANSPORT	281	3.43E-02
GO_POSITIVE_REGULATION_OF_PROTEIN_BINDING	67	3.44E-02
REACTOME_GLYCOLYSIS	24	3.44E-02
GO_POSITIVE_REGULATION_OF_NEURON_DIFFERENTIATION	258	3.50E-02
GO_BASEMENT_MEMBRANE	78	3.50E-02
GO_RESPONSE_TO_NICOTINE	32	3.51E-02

GO_NAD_METABOLIC_PROCESS	41	3.51E-02
GO_MIDGUT_DEVELOPMENT	12	3.52E-02
GO_REGULATION_OF_TRANSCRIPTION_INVOLVED_IN_CELL_FATE_COMMITI	16	3.52E-02
GO_ODONTOGENESIS	85	3.53E-02
GO_LOW_DENSITY_LIPOPROTEIN_RECEPTOR_ACTIVITY	10	3.54E-02
PID_INTEGRIN_A9B1_PATHWAY	17	3.56E-02
GO_SECRETORY_GRANULE_LUMEN	42	3.56E-02
GO_POSTSYNAPSE	289	3.57E-02
GO_CELL_SURFACE_RECEPTOR_SIGNALING_PATHWAY_INVOLVED_IN_CELL	45	3.64E-02
GO_CIRCULATORY_SYSTEM_PROCESS	242	3.67E-02
GO_RESPONSE_TO_PROSTAGLANDIN	23	3.73E-02
GO_PHASIC_SMOOTH_MUSCLE_CONTRACTION	10	3.73E-02
GO_CELLULAR_RESPONSE_TO_CADMIUM_ION	7	3.73E-02
GO_POSITIVE_REGULATION_OF_STEROL_TRANSPORT	15	3.73E-02
GO_ADENYLATE_CYCLASE_ACTIVATING_G_PROTEIN_COUPLED_RECEPTOF	36	3.74E-02
GO_DEVELOPMENTAL_MATURATION	142	3.74E-02
GO_NEUROPEPTIDE_BINDING	10	3.74E-02
GO_PANCREAS_DEVELOPMENT	59	3.80E-02
GO_CARDIAC_MUSCLE_CELL_CONTRACTION	22	3.84E-02
GO_POSITIVE_REGULATION_OF_AMINO_ACID_TRANSPORT	10	3.86E-02
GO_POSITIVE_REGULATION_OF_NEURON_PROJECTION_DEVELOPMENT	207	3.86E-02
GO_AMIDE_TRANSMEMBRANE_TRANSPORTER_ACTIVITY	10	3.87E-02
GO_SYNAPTIC_VESICLE_RECYCLING	22	3.89E-02
GO_SODIUM_CHANNEL_ACTIVITY	21	3.89E-02
GO_EMBRYONIC_MORPHOGENESIS	458	3.98E-02
GO_ENDOCHONDRAL_BONE_MORPHOGENESIS	41	3.98E-02
GO_ADRENAL_GLAND_DEVELOPMENT	18	3.98E-02
GO_ATRIOVENTRICULAR_VALVE_MORPHOGENESIS	15	3.99E-02
GO_CELLULAR_RESPONSE_TO_VASCULAR_ENDOTHELIAL_GROWTH_FACTO	27	4.00E-02
GO_CELL_SUBSTRATE_ADHESION	136	4.02E-02
GO_POSITIVE_REGULATION_OF_PROTEIN_TYROSINE_KINASE_ACTIVITY	27	4.03E-02
GO_CELL_SURFACE	458	4.04E-02
REACTOME_FACILITATIVE_NA_INDEPENDENT_GLUCOSE_TRANSPORTERS	9	4.10E-02
GO_DENDRITE_DEVELOPMENT	67	4.11E-02
GO_MONOVALENT_INORGANIC_CATION_TRANSMEMBRANE_TRANSPORTER	227	4.13E-02
GO_REGULATION_OF_SYNAPSE_ASSEMBLY	50	4.23E-02
GO_HINDLIMB_MORPHOGENESIS	32	4.24E-02
KEGG_ASTHMA	13	4.27E-02
REACTOME_NEURONAL_SYSTEM	192	4.27E-02
GO_APOLIPOPROTEIN_BINDING	11	4.27E-02
GO_INORGANIC_ION_TRANSMEMBRANE_TRANSPORT	380	4.27E-02
GO_METENCEPHALON_DEVELOPMENT	79	4.39E-02
GO_SERTOLI_CELL_DIFFERENTIATION	13	4.39E-02
GO_HINDBRAIN_DEVELOPMENT	109	4.40E-02
GO_METAL_ION_TRANSPORT	376	4.41E-02
GO_HORMONE_ACTIVITY	36	4.41E-02
GO_RECEPTOR_SERINE_THREONINE_KINASE_BINDING	11	4.42E-02

GO_PHOSPHOLIPASE_C_ACTIVATING_G_PROTEIN_COUPLED_RECEPTOR_S	42	4.42E-02
KEGG_ALLOGRAFT_REJECTION	19	4.42E-02
GO_RESPONSE_TO_GROWTH_FACTOR	384	4.45E-02
GO_REGULATION_OF_ENDOTHELIAL_CELL_PROLIFERATION	77	4.46E-02
GO_UROGENITAL_SYSTEM_DEVELOPMENT	247	4.52E-02
GO_REGULATION_OF_METAL_ION_TRANSPORT	214	4.53E-02
GO_MOTOR_NEURON_AXON_GUIDANCE	27	4.53E-02
GO_REGULATION_OF_PROTEIN_BINDING	152	4.54E-02
GO_SERINE_TYPE_ENDOPEPTIDASE_INHIBITOR_ACTIVITY	36	4.55E-02
GO_ARTERY_MORPHOGENESIS	43	4.56E-02
GO_REGULATION_OF_SYNAPSE_STRUCTURE_OR_ACTIVITY	165	4.60E-02
GO_NEGATIVE_REGULATION_OF_SMOOTH_MUSCLE_CONTRACTION	14	4.61E-02
GO_MESENCHYMAL_TO_EPITHELIAL_TRANSITION	12	4.61E-02
GO_RESPIRATORY_BURST	7	4.61E-02
GO_SODIUM_ION_TRANSPORT	88	4.68E-02
GO_PROTEIN_FOLDING_IN_ENDOPLASMIC_RETICULUM	8	4.68E-02
GO_CHROMOCENTER	13	4.68E-02
GO_REGULATION_OF_ODONTOGENESIS_OF_DENTIN_CONTAINING_TOOTH	8	4.69E-02
REACTOME_SEMAPHORIN_INTERACTIONS	59	4.69E-02
GO_URETER_DEVELOPMENT	11	4.70E-02
GO_PROTEIN_LIPID_COMPLEX_BINDING	13	4.76E-02
GO_PLATELET_DERIVED_GROWTH_FACTOR_BINDING	9	4.77E-02
GO_SYNAPSE_ASSEMBLY	47	4.78E-02
GO_CYCLIC_NUCLEOTIDE_MEDIATED_SIGNALING	33	4.78E-02
GO_ION_CHANNEL_BINDING	91	4.78E-02
GO_PROSTATE_GLAND_MORPHOGENESIS	17	4.81E-02
GO_TRANSPORT_VESICLE_MEMBRANE	116	4.81E-02
REACTOME_AXON_GUIDANCE	215	4.81E-02
GO_REGULATION_OF_AXON_GUIDANCE	34	4.87E-02
PID_BETA_CATENIN_NUC_PATHWAY	68	4.87E-02
GO_POSITIVE_REGULATION_OF_ENDOTHELIAL_CELL_PROLIFERATION	55	4.91E-02
GO_COCHLEA_DEVELOPMENT	28	4.96E-02
GO_REGULATION_OF_OSSIFICATION	137	4.97E-02
GO_VENTRICULAR_SEPTUM_MORPHOGENESIS	25	4.99E-02
