# Supplementary material for: CYP2J2 Modulates Diverse Transcriptional Programs in Adult Human Cardiomyocytes
Source: Sci Rep. 2020 Mar 24;10:5329. doi: 10.1038/s41598-020-62174-w (PMC7093536; doi:10.1038/s41598-020-62174-w)
Supplement: Supplementary file 3 — Supplementary table S3 [file 41598_2020_62174_MOESM3_ESM.pdf]

Table S3. List of leading edge genes for each biological module from gene set enrichment analysis.

Ion Channel Module	Development Module	ECM Module	Metabolism Module
GNG5	MAPK14	ITIH4	PPP2CB
OAZ3	MYO1C	BDNF	GPI
KCNB2	EGR3	EGF	ENO2
PDE6G	GNG5	SEMA6C	PGK1
SLC12A2	HSPB1	MST1L	ALDOA
SLC24A2	PLXNA4	THPO	ENO3
SLC22A1	DCT	TNF	PGAM1
CPEB4	PRKD2	LMAN1	ALDOC
STIM1	FLT3	MAPK1	HK1
GRM8	NOTCH2	GPC4	HK2
SLC6A16	ALCAM	FREM2	LDHB
HVCN1	ELL3	TYMP	QPRT
B2M	NOTCH4	WT1	NADSYN1
SLC6A15	GATA2	NFKB2	PNP
PDE11A	WNT9A	ITGB4	AFMID
SLC9A2	GNAT2	LGALSL	GPD1
SIX4	KNDC1	FOXC2	GPD1L
NTRK2	NLGN2	PLXNB1	PFKM
FAM26E	PGAP1	SEMA4A	OGDHL
SYT9	ERN1	SH3PXD2B	HOGA1
ATP6V0E1	CDX2	ONECUT2	PDHB
BDNF	HOXA3	SOSTDC1	DLAT
CLSTN3	TLX3	CDON	PGAM4
ATP6V1B1	LRP5L	ELF3	ACSS1
SLITRK3	BRSK1	ANXA6	ALDH3A1
HOMER3	SPTBN5	MYH11	ALDH7A1
CELSR2	WEE1	CFP	ALDH9A1
TRPV3	HOXA6	PAK1	
ANO1	ZSWIM6	GPI	
FLRT3	SHROOM2	PTGES	
DAGLA	SEMA6C	SEMA6A	
PRKX	AKIP1	DBI	
ATP5S	MLLT3	MME	
SLC23A2	FAM20C	SEMA6D	
NNT	TUFT1	MGST1	
LMAN1	INSR	ICAM4	
EGR2	MSLN	LRP1	
VAMP1	UBB	RASL11B	
NTRK3	TFEB	PDGFRA	
AMHR2	BARHL2	CHST15	
MAP2K1	HPSE	ATP7A	

SLC22A18
RASD1
NIPSNAP1
COL4A1
SIX1
HSPA1A
PRIMA1
PHKG2
PPP2R5E
PPIB
FSD1
EPOR
VEGFA
HSPA4
ATG5
AMIGO2
COL9A1
MRAP2
EPHA5
IRS1
IL11
CD19
PLA2G4B
CDON
MASP2
CDK5R2
EFEMP2
SLC25A3
DLK2
FSTL4
EGFLAM
GGT7
PDIA4
PRKCSH
ATP6V1G2
PAK1
GCNT4
LRRC8C
PSME2
IL17RB
S100A2
ROBO3
CD274
IRS2
ZNF281
RCC2
SIGLEC1
ACTL6A
PAK6
MIXL1
FREM2
CMTM7
ESRRB
TYMP
TFAP2C
L3MBTL3
CSNK2A1
DHX36
AMBN
PPAT
PDLIM7
AP1G1
BCAN
SEZ6L
ATG5
AEBP1
IFITM1
KDM1A
HMGCS1
ONECUT2
DUSP2
NPY
DOCK1
NSDHL
CYP24A1
ERCC3
SCN4A
PSAP
GPI
PLCD1
DNAI1
MID1
ALOX15
CRELD1
DNM1
DAAM2
PRKCH
MYO10
GPM6B
KDR
CSGALNACT1
IL32
HSP90B1
SEMA3F
CTSL
F3
FZD8
ZMPSTE24
P3H1
ELFN1
MAGEE1
CHST11
FAM20C
HYAL1
SULF1
CBS
HAS3
ADAM32
SEMA6B
ZEB1
ADAMTSL5
P4HA2
PLAT
GSTM2
GSTM4
TPM2
AMBN
PPIB
MYO1E
PEBP1
ELFN2
MANF
NOD2
PRRX1
KIF9
BAMBI
NTNG1
ONECUT1
MASP2
SEMA7A
ID2
VWA7

SMAD2	APOLD1	ITGB8
KLF15	DLL4	ICAM5
NXPH4	ALDH9A1	NKX2-1
CRELD1	ADAMTS4	ECEL1
SEMA6A	G6PD	S100A2
BOC	MME	HPX
AANAT	MTPN	RAMP2
CLDND1	MGST1	SMAD2
WDR73	COL6A2	TMEM184A
PEX12	LRP1	CRELD1
MATN1	POLR2A	TBX20
EFNA1	S1PR3	PHOSPHO1
EHD3	VWA2	PLXNA2
LTBP1	LYPD3	ALPL
ALDH9A1	COL7A1	CALU
FOXD1	ST8SIA4	DACT1
HOXB2	ENPP2	CREB3L1
ATL1	CXCL2	OTOG
NCAN	IPMK	FOXD1
EFNB1	HRH1	HPSE
EGFL8	HRH2	LGALS1
GDPD5	HIF1A	GPC1
NECAB2	LAMC3	HSP90AB1
RAB26	GPM6B	GAL3ST4
S100A3	NEK3	EGFL8
KATNB1	EPDR1	SSPO
VWA2	ARHGEF26	MUC1
CTF1	PDE4B	MST1
GTF2I	CDKN1A	S100A3
COL7A1	MN1	CTF1
COL3A1	MDGA1	LYPD3
TP53	DFNA5	TGM1
AMH	FGFR4	CXCL2
TCHH	CDKL5	SGCB
SCUBE2	UNCX	TCHH
HCST	PLP2	FRZB
KCNK5	BTK	SEMA3G
TAP1	FOXL1	SCUBE2
PTP4A3	ESR2	ATP1A3
NEK3	COLQ	SYNJ2BP
EPDR1	CLSTN3	NELL1
TRPM4	POLR2K	S100A1
SYNJ2BP	AQP6	VWA3A
NELL1	IL1R1	LGALS3BP

PLCH2	DMRTA1	AEBP1
LRP10	LDB1	CNTF
LGMN	LTB4R2	VWCE
EFHD1	CACNA1G	IL11
DAB2	NFIA	C1QL4
LRP6	ARF4	MYLK
SLC41A1	CGN	MRC2
VWCE	PREX1	PCSK5
EXOC3L1	SNCA	ZNF423
HSP90B1	THBS4	PLXNC1
TRPC3	PABPC1L	MUC3A
TMEM100	CCR10	PMP22
MICA	CACNA1I	OGDHL
COL24A1	FGF13	MEGF10
FOXL1	ADRA2C	RNF165
EHD2	ARNTL	ITGA7
FEZ1	MEIS1	TGFB1I1
AXL	PLA2G6	RECK
SHROOM2	SULF1	HSPA5
F3	USP2	SV2A
CAPN12	AQP3	CDKN1C
ATP2A2	ACTG2	C17orf58
NFIA	TNF	C1QTNF6
FGF8	PTCH2	NOG
ELFN1	SHC1	CITED1
STMN3	ZFYVE9	PLXNB3
DACH1	SGCE	HTRA3
COL25A1	CXCL1	BRINP2
PPP2R5A	AMHR2	CBLN1
NCOA1	RAP1GAP	CITED2
MYOD1	TTPA	SNED1
COL16A1	EXTL1	LOXL3
TNS1	MSH4	CSPG4
VAMP2	CHGA	WNT10B
STAB1	TGM2	GATA6
PRKG2	TTYH1	AMH
OPA1	GFRA2	SNAI2
RAB6A	EXT2	SOX9
BARX1	IGFBP4	MAP1A
TIMM17A	RPS6KA2	LGI3
PSMB9	OTOF	CRLF1
ADAMTS2	PPIB	DKK3
AQP3	SPOCK1	FNDC1
RPH3AL	HPCA	SCUBE3

PTCH2
MAPK1
ITGB1BP2
ADAMTS14
KIF1A
PADI3
ABCG1
BMP6
ERBB3
CASP10
COL9A2
MICB
RAP1GAP
SYNRG
PLEKHG2
LIF
TGM2
ATP6AP1
ITGB4
SERPINH1
TMEM145
RSPO2
SNED1
CHST4
ETV1
BCAN
ADAM21
GUCY1A3
COL5A1
CSPG4
PDE1C
PLXNB1
ALOX15B
ADD2
NOD2
TCN2
NIPAL3
MMP25
CPT1C
NPNT
CISH
ESPN
SDC1
GATA6

SNED1
DGAT2
CCDC151
ALOX15B
MBNL1
ARC
MYO1G
AMIGO2
BAMBI
DDIT4
SCN3B
SYNGAP1
DUSP15
CNTFR
ONECUT1
SOSTDC1
ADRB2
ABLIM3
HBEGF
EIF2B4
CDK5R2
CBL
REC8
ASXL1
HAPLN3
DOCK4
ACTL6B
IL6R
MYH11
SYNDIG1
GCNT4
SLIT1
KCNIP2
TPBG
ITGA10
ITGA9
LTK
ITGA11
FBXO38
ACAN
SIX4
SF3A2
FGF12
ITGA7

FGFR4
ANGPTL6
ITGA11
GDNF
CRIP2
CEL
VWA5A
FGF8
FLRT1
ITGB7
NTNG2
FGF13
SCUBE1
IGFBP5
LAYN
IGFBPL1
PBLD
NRP1
ACVRL1
WNT6
LIF
IGFBP4
COL4A1
SVEP1
MEGF11
PODNL1
BCAN
ADAM21
MMP25
MSX2
EMILIN1
GPC3
CILP2
EFEMP2
ADAMTS18
TGFBR3
SNCA
GPC2
ADAMTS10
ITGA9
MMP24
CTSV
MMP14
CHRDL1

EFCAB10	TGFB1I1	PLAU
SEMA7A	EGF	ITGB3
AAAS	CTNNA1	WNT3A
MEX3B	HYAL1	COL9A1
PDPN	AVIL	MMP16
AOC3	DACT1	DNAJB6
RGS4	ID3	CXCL1
KDM5B	NAP1L2	TGM2
PLCD4	BTG1	MATN1
HMCN2	IST1	BMPER
FKBP7	NCAN	EMILIN2
SLC47A1	ADRA2B	EGFLAM
PSAP	FLT4	LTBP1
MYH11	OPRD1	DAB2
REPS2	LFNG	DPYSL3
ECEL1	SORBS1	ADAMTS4
ABCB6	CNTNAP2	CPAMD8
ADAMTS1	CACNA1C	CRTAC1
GPI	FGF5	RELL2
PLCD1	FOXE1	CRISPLD2
NID1	SLC17A7	VEGFA
KCNF1	SGCB	ITGA3
TPBG	PDE3A	VWA2
BMP1	EXOC4	FGF5
ITGA9	METRN	INHA
SPOCK3	BECN1	SDC1
MMP24	LINGO1	ADAM11
FBLN2	FKBP1A	MMP11
NOTCH3	IGSF9	CXCL12
PRKAA2	SLC24A4	FKBP1A
INHBB	C3AR1	INHBE
COL15A1	PAPSS2	HMCN2
MMP14	MTF2	ADAM12
KCNAB2	FBLN5	LAMA2
RASGRP3	SCN2A	SHH
ITGA11	SRCIN1	HMMR
AQP4	EPOR	SSC5D
BMP3	RPS6KB1	ITGA10
PDIA3	ZFP36L1	MFAP2
RDH11	BCOR	PDGFB
INHA	YWHAB	SUSD5
MXRA8	SLC39A5	NCAN
COMP	SND1	NRP2
MMP11	SH3PXD2B	NPNT

PRKCH	CNTF	TLL2
PITX3	SYT3	ADAMTS17
HPCAL4	ECM1	SERPING1
ESAM	LRP6	ADAMTS5
CALML6	RASIP1	SULF2
FLRT1	ITPKA	CCDC80
NTNG2	DLX3	RSPO2
ADAMTS9	BCAR3	WNT5A
SLC26A10	NTNG1	LOX
IGFBP5	MYLK	PXDN
CALU	FANCF	TIMP3
HOXA1	KIAA1217	IGF2
GNRHR2	DDX21	ADAMTSL3
NDFIP2	EHD2	COLEC12
NGEF	PIP5K1A	TGFB3
ALDH5A1	ASPH	OLFML2A
DPYSL3	RIMS2	COLQ
CD44	DACH1	SPON1
NIPAL4	SLITRK3	SPOCK3
CD24	CCND1	SPOCK1
SCG3	CELSR2	LAMC3
GNRH2	SPINT1	FST
LAYN	KDM6A	MMP17
ADAMTS18	BARX1	ADAMTS9
MTPN	PRKX	NAV2
KDM4A	PRKCI	BMP6
TMEM30A	TYRO3	KCP
ADAM9	NR4A2	MDK
GPR157	LAMA1	COL24A1
CD1D	INHA	HBEGF
GAL3ST4	TCF7L2	TFPI2
PPP2CB	HSPA5	PTCH1
PDZRN3	CTR9	ENG
NIPA1	COL9A2	COL25A1
COL6A2	PTGER4	ADAMTS7
CAPN5	HOXD1	ADAMTSL4
RENBP	PITX3	BMP1
ADAMTS7	TRIM45	ADAMTS20
FKBP10	CAMK1D	GDF11
SLC5A5	FERMT3	SPOCK2
FGF5	TMEM107	ADAM19
LHX1	PLK5	ADAMTS2
PALMD	ITGB4	ITGA8
ENPP2	NTRK2	FBN3

CELF4	ATL1	FGF12
SLC12A9	NDEL1	ADAMTS14
THRA	PAK2	CHRD
FBXO2	PREX2	APLP1
FBN3	BDNF	HAPLN3
ATP6AP1L	MAP6	CD44
RTN2	MMD	SERPINH1
ZIC1	COL9A1	ADAM9
FRZB	GTPBP4	COL6A6
SVEP1	PAX3	COL6A2
PGF	KATNB1	FBLN2
CCDC47	NES	INHBB
RPS6KA2	WASF3	BMP3
ARHGEF9	PLAG1	COL16A1
COL27A1	PLD6	ADAMTS16
SLIT3	BRSK2	PGF
GRIP2	CCNE1	WNT2B
FBLN5	DCLK1	SERPINE2
BEST3	CREB3L2	COL7A1
TBX3	ITGB8	LAMB1
ARHGAP32	MMD2	COL9A2
COX6B2	CRLF1	ACAN
PCOLCE2	LRRN2	SFRP1
TM2D1	RGS20	CRIM1
HMMR	RASAL1	GDF7
EPHB1	CUX1	CCBE1
SLC44A1	POLR2C	FBLN5
YWHAB	GFRA1	COL3A1
LSAMP	KDR	ECM1
MYBPC2	TBC1D7	ACHE
MMP16	SRGAP2	GDF6
ATP6V1C2	INHBB	DAG1
WNT10B	DAB2	ADAMTS1
FADS1	LMO4	THBS4
TLL2	HAP1	SLIT3
VCAN	PLAU	EFEMP1
COL11A2	SPNS2	BMP2
PLS3	CPNE5	NID1
WNK4	CD72	SLIT2
SFXN1	THRA	COL15A1
RNF6	NID2	COL6A1
AQP6	KLF15	PCOLCE2
AHNAK	DISC1	SPARC
ITGB8	HES7	COL13A1

ACTN1	ACTN1	VCAN
TAP2	POU4F2	COL27A1
CCDC78	SMARCC2	COMP
TGFBR3	SBDS	FBN1
BTG2	ETV1	MMP2
ADRA2C	BTG2	COL5A1
EFEMP1	ITGB7	LAMA1
LAG3	MYOD1	TNC
CAD	OLIG1	COL11A2
NDRG4	ATP6V1B1	NID2
CCBE1	IGFBP5	FN1
DBN1	PTH1R	COL2A1
NKD1	KCND3	COL14A1
TAL1	CREB3L1	
FBN1	OPA1	
FN1	FLRT3	
APOL1	SSBP3	
AHNAK2	EPHA5	
SLC27A6	WNT10B	
DGKD	ADAM9	
HSPA1B	CUX2	
MAP3K12	HSP90AB1	
TNF	CAPN5	
RORB	ROCK2	
USP14	PCDH8	
MERTK	SHCBP1	
PROCR	RGMB	
ITGA7	UNC5B	
ARHGEF17	TUBB3	
MYOM2	FOXB1	
MDK	MAP2K1	
VN1R1	KCNC1	
MYO15A	RAB3A	
DNAJC6	LAMA2	
SMAD5	VAX1	
CADM2	AMH	
APLP1	SYP	
ARHGEF6	TMIE	
ABCA1	COL4A1	
MCAM	KIF5C	
OLFM1	CCDC47	
CRTAC1	ZC4H2	
GCLC	HHEX	
CAMKK2	CBLN1	

DDN
BAK1
PRKD1
LRP12
TRHDE
NGFR
COL6A1
CD8A
SEZ6L
AP3B2
ACVRL1
ARHGEF2
GLI1
RGS11
LDLR
CDH7
TMEM206
NEFH
PACSIN1
COL2A1
TPRG1L
WASF3
PDGFRA
HLA-A
MMP17
CNTFR
APC
NFATC4
CBL
OXGR1
GPR3
ANO8
PAX5
GPM6B
RASL10B
BEST2
UNC13D
SLC30A10
POU4F1
IL6R
ABCB4
RDX
HLA-DOA
RGS7
PLEKHA1
CHRNA10
BOC
OTX1
GPC1
EGR2
ACHE
MRC2
SS18L1
WNK4
LRRC17
PTPN5
TGFBR3
EFNB3
KDM5B
PRKD1
S1PR1
DPYSL4
COL25A1
CA2
NID1
NKD1
BMP1
IGF2
ROBO3
NPTN
COL3A1
DNAAF1
CHRDL1
FNDC3A
RYR1
SEMA6B
RAMP2
CORO1A
SCN5A
SLC9A6
ERBB3
LHX6
PRKCQ
NTRK3
USH1G
ATP7A
ATOH8
CNTNAP1

ITGA10
CD33
STRA6
EN1
SLC39A5
SLC39A6
SCUBE3
TBX2
TTYH2
DCHS2
PLAU
SPNS2
CD55
MMP2
NID2
SLC4A11
DISC1
ANO4
PPFIA4
EPHA7
GPRC5A
PCDHA10
NEFM
GPR83
PCSK5
CABP4
RTN3
PREX1
KCNB1
THBS4
PCDH10
SCUBE1
PREX2
ATP7B
GM2A
LGR5
HOMER2
OTOG
ITPKA
SNAP91
DGKA
TNFRSF21
USP2
LRP2
NTNG2
INSM1
BMPER
NAV2
ZIC1
LGR5
MAN2A1
SHANK3
ACTN2
TP53INP2
CLSTN1
MYO1E
TMEM30A
PAK1
TSHZ1
GPRC5B
LOXL3
COL5A1
MICAL2
COL6A1
ITGA3
EEF2K
SDK2
GDPD5
ETS2
NEFH
PACSIN1
ETV4
NCOA1
KLF7
APC
FZD4
CABP4
MSN
NFATC4
SLC9A3R1
CCDC40
MAPK8IP2
RORB
SLC11A2
HOXA7
RECK
LAMB1
FUZ

TGFB3	MFAP2
CUX2	EN1
ITGB3	PTF1A
GPR50	CUL7
TUBB4A	SEMA6D
NPY1R	RAB10
NPTX1	CDKN1C
RIN1	PKDCC
LRP1	CDK5R1
SPARC	SGK1
NRP1	DRD2
SNTB2	VCAN
FOXN4	COL11A2
RASGRP1	SCARF1
RAB21	LRP8
ZNRF2	PLS3
COL13A1	AXL
EPHX2	RERE
MAP1B	PCSK5
PCDH18	CITED2
SLC5A6	AMOT
ANXA6	LRIG3
LAMA2	MATN1
SYNDIG1	TSPAN12
TPM2	ROM1
ATP6V1B2	ALPL
PCDHGA11	SEMA4A
PIK3R5	RASSF2
ZC4H2	PAX5
KALRN	FOXJ1
NOL3	PDZD7
FAT3	EPHA8
RGS20	CHD5
SLC2A4	PTPRM
SRCIN1	EFNB1
SPOCK2	ZMYND8
PDE1A	UNC13A
SLC10A4	OLIG2
ADORA1	ITGB3
RRH	PTPRD
SYNGAP1	SYT17
SERPING1	NPTX1
CEL	EPHA7
EEF2K	ACVRL1

ITSN1	SERPINE2
JPH1	MYO15A
SLC9A3R1	PITX2
SDK2	DAB1
IL1R1	MAGED1
ATP8A2	SEMA3G
LGI3	NOTCH3
KCNK7	MKS1
ARF4	NGFR
EFNB3	WDR19
DLL1	MMP16
DOCK4	FLRT1
SHANK3	MAPK1
TNFRSF10B	NPNT
COL14A1	HOXC11
ADIPOR2	PHOSPHO1
HLA-DRB5	SDC1
ADAMTS16	LATS2
WNT6	FOXC1
DNER	PAX1
SORCS1	FGFR1
MR1	DLX6
NEFL	SUFU
SLC31A1	DPYSL3
CXCL2	CD44
PDE3A	NLGN3
PMP22	BAK1
NPTX2	INSIG1
SGCE	LHX9
TMEM63C	SPARC
CDK5R1	NDRG4
PCDH1	VLDLR
PCDHGA7	SERPINH1
PRKCQ	CSGALNACT1
ATP7A	CDON
STIM2	KIDINS220
ITGB7	DMRTA2
TBX20	RNF165
SH2D5	SEMA6A
SLIT2	SPTBN4
GBX1	FEZ1
DLGAP3	MEGF11
SLC18A3	BCL2L11
NKAIN1	DNAJB6

NSF	ADCYAP1R1
MME	POU4F1
RASD2	LIF
CDH15	SMAD5
HPCA	HOXC4
CHD7	CRABP2
SHH	SNX3
SCAMP5	PROX1
CLCN5	FRZB
CDH22	ADAMTS1
GRID1	PBX3
ITGA3	RDH10
ARC	PLXNB1
KCNH3	ADAMTS7
SORBS1	BMP3
HLA-B	EFNA1
TPCN1	LHX2
TRO	RAB21
MAGEE1	HOXA1
FGF13	CHST11
L1CAM	PDGFB
DGKG	RIPPLY2
UBASH3B	HOXB5
PCDHGC4	HOXD13
HOPX	COMP
SLC13A3	TGFB3
HSPA2	NEFL
P2RY4	MED12
ENG	MERTK
RASA3	RDX
RHOA	GPC3
DPP4	HOXB6
RET	CD24
C3AR1	HOXB2
MAOA	HOXB4
KCTD16	MMP14
PTGFR	NKX3-2
LRRC4	NKX6-2
SNCAIP	GATA6
NKX2-1	MAF
SHC1	SMAD2
PDE4C	ZIC3
ROCK2	GDF6
SLC4A5	CXCL12

RPS6KB1	MAP1B
VLDLR	SYT2
CXCL1	SOX2
MPDZ	HOXB9
SLC4A9	PTPRO
SYT3	COL27A1
THBD	TMEM100
KCTD12	GLI1
COLQ	TAL1
HLA-DPB1	KCNA2
GPHN	CYP26B1
RASGRP2	ATP8A2
FZD8	GBX1
P2RX6	TBC1D20
AMOT	RARB
BAIAP3	NTRK1
NTRK1	RBFOX2
TNNI3	EOMES
TNNC1	WT1
NAV2	CHD7
PTH1R	WNT6
ADORA2B	PDGFRA
PTGER2	LHX3
SYPL2	EDNRB
IGF2	EPHB1
SFRP1	NKX2-2
ATP2A3	SHANK1
DSP	SOX4
APLN	TBX2
HBEGF	OSR1
CABP1	SEMA3F
FXYD6	FOXC2
HOXD9	RSPO2
PTPN5	GDF11
HSPA5	HAND1
DES	CHRD
DNM3	HOXB3
THRB	DLX1
DSC2	COL13A1
SGCB	L1CAM
DAB1	HOXD11
AKAP9	SULF2
SYNGR1	NFASC
PRKCG	SLIT3

SLC39A11	VEGFA
YWHAG	OLFM1
IGSF9	MMP2
AVPR1A	STRA6
SSTR1	ZEB1
SPOCK1	TFAP2A
PLCB2	FN1
CORO1A	ITGA8
TNC	FOXN4
CACNA1C	FOXD1
DNM1	DAG1
C2CD4C	TNC
PTPRO	SEMA7A
AMPH	ID2
NAAA	ENG
ATP5F1	HOXD9
MC1R	CITED1
PLCL1	PLXNA2
S100A1	PRRX1
PPFIA3	DMRT3
RIMS2	SPTBN1
CDHR1	EFEMP1
SYTL4	HOXB8
HOXB8	NRP2
S1PR3	NRXN3
NMUR2	FBN1
CNGA4	SIX1
PCDHB2	RARG
PCDH19	PGF
UCN2	BMP6
PCDHGC3	HOXA2
HTR6	PLXNC1
CALCB	ADAMTS16
TTYH1	NKX2-1
HOXD10	DLL1
GPRC5B	PAX7
CCR10	SCN1B
HSP90AB1	LRP4
GABRE	MYC
WNT5A	CRMP1
LPAR2	RHOA
CLCN1	RET
PROKR1	SHOX2
NPR1	WNT3A

CBLN1
GRIK3
GIPR
CANX
BMP2
CHRNA10
PDE2A
NPY
ZMYND8
PGM5
WNT3A
GRIK4
TBXA2R
LTB4R2
GNG2
C2CD4A
SLC9A5
ID2
DCHS1
GSTM2
SLC6A17
SYT14
DMRT3
FZD10
AUTS2
SLC11A2
VDAC2
PTGER4
SULF2
ADCY5
CACNG6
P2RY11
SHISA7
SLC9A6
HTR2C
SYTL2
PDGFB
GNB3
CA2
PVALB
GRIK5
GRM2
FKBP1A
WNT2B

HEY1
SALL1
DLX5
PLXNB3
TBX3
GDNF
GDF7
SFRP1
TBX20
SLIT2
HOXA11
VANGL2
SHH
PAX6
HOXD10
SOX8
NRP1
EZR
WNT5A
WNT2B
SIX2
FGF8
COL2A1
SNAI2
MSX2
NOG
PTCH1
DCHS1
LHX1
BMP2
PAX2
SATB2
SOX9

RYR1
PTPRD
HLA-DMB
PCDH8
ATP2B3
ATP4A
SYNGR4
PPP3CA
NOS2
KCNV1
IGF2BP1
SLC30A3
FZD4
LRP8
SERPINE2
SYT11
HERPUD1
PBX3
SYN1
SYT16
LTB4R
SHANK2
PLCG2
DYSF
CALCRL
OTOF
CNGA1
PLA2G6
VDAC1
RAB3A
ADRA2B
ITGA8
GABRD
KCNA3
GCGR
ACTN2
HRH1
ARL6IP1
ADCY7
RAMP2
SLC6A1
CCKBR
MAPK8IP2
SYT5

CHGA
SCN4A
TRPV4
BSN
HRH2
ADRA1B
MYLK
GDNF
KCNN3
CPLX1
SGK1
NFASC
PTCH1
SV2A
NMUR1
S1PR1
RAB3B
KCNC4
SYT12
HLA-DMA
PDE1B
EZR
UNC13A
SPTBN1
HAP1
SYN3
NPY5R
ACHE
PDE4B
SPTBN4
SLC8A3
LRP4
CHRM4
GABRQ
NPTN
STX1B
SLC8A2
DAG1
DOC2A
SLC24A4
ATP2A1
NRXN2
CNTNAP1
KCNN2

YWHAE
HLA-DRB1
CACNA2D2
GRIN3B
NLGN3
SLC17A7
CLSTN1
SYT6
DGKI
CNTNAP2
OPRL1
GRM4
CACNA1I
TRPM5
SYT7
SHANK1
ADRB1
CXCL12
SYT17
GABBR2
SLC38A3
RASGRF1
FGF12
KCNA7
GNAO1
ANK2
CAMK2D
KCNJ12
KCNH2
KCNAB3
SNCA
CACNA2D1
CACNA1G
CACNG8
ASPH
GNG4
SCN4B
CACNG7
SYP
HMOX1
KCNJ10
SNAP25
P2RX2
EDNRB

GPD1L
SLC13A4
OPRD1
SSTR2
CHRNA4
KCNIP3
KCND1
DRD2
KCNIP2
NRXN3
CHRNA3
KCNC3
HCN4
ADRB2
KCND3
ABCC8
SCN3B
ATP1A3
SCN2A
KCNMA1
SYT2
ATP1B2
ADCYAP1R1
ABAT
KCNC1
SCN5A
KCNA1
SCN1B
CACNA1B
KCNA2
