# Supplementary material for: CYP2J2 Modulates Diverse Transcriptional Programs in Adult Human Cardiomyocytes
Source: Sci Rep. 2020 Mar 24;10:5329. doi: 10.1038/s41598-020-62174-w (PMC7093536; doi:10.1038/s41598-020-62174-w)
Supplement: Supplementary file 4 — Supplementary table S4 [file 41598_2020_62174_MOESM4_ESM.pdf]

Table S4. List of "Ion Channel Signaling" interaction network members upregulated in CYP2J2-silenced cardiomyoctes.

Gene Symbol	Gene Name	Location	Family
ASPH	aspartate beta-hydroxylase	Cytoplasm	enzyme
ATP1A3	ATPase Na+/K+ transporting subunit alpha 3	Plasma Membrane	transporter
ATP1B2	ATPase Na+/K+ transporting subunit beta 2	Plasma Membrane	transporter
ATP5PB	ATP synthase peripheral stalk-membrane subunit b	Cytoplasm	transporter
CA2	carbonic anhydrase 2	Cytoplasm	enzyme
CACNA1B	calcium voltage-gated channel subunit alpha1 B	Plasma Membrane	ion channel
CALU	calumenin	Cytoplasm	other
CAMKK2	calcium/calmodulin dependent protein kinase kinase 2	Cytoplasm	kinase
CANX	calnexin	Cytoplasm	other
CHRM4	cholinergic receptor muscarinic 4	Plasma Membrane	G-protein coupled receptor
CNTNAP1	contactin associated protein 1	Plasma Membrane	other
CNTNAP2	contactin associated protein like 2	Plasma Membrane	other
CPLX1	complexin 1	Plasma Membrane	transporter
CRTAC1	cartilage acidic protein 1	Extracellular Space	other
DACT1	dishevelled binding antagonist of beta catenin 1	Cytoplasm	other
DAG1	dystroglycan 1	Plasma Membrane	transmembrane receptor
DKK3	dickkopf WNT signaling pathway inhibitor 3	Extracellular Space	cytokine
EFEMP1	EGF containing fibulin extracellular matrix protein 1	Extracellular Space	enzyme
EZR	ezrin	Plasma Membrane	other
FBLN5	fibulin 5	Extracellular Space	other
FBN1	fibrillin 1	Extracellular Space	other
FGF12	fibroblast growth factor 12	Extracellular Space	other
GABBR2	gamma-aminobutyric acid type B receptor subunit 2	Plasma Membrane	G-protein coupled receptor
GFPT2	glutamine-fructose-6-phosphate transaminase 2	Cytoplasm	enzyme
GNAO1	G protein subunit alpha o1	Plasma Membrane	enzyme
HAP1	huntingtin associated protein 1	Cytoplasm	other
HSPA5	heat shock protein family A (Hsp70) member 5	Cytoplasm	enzyme
ITGA8	integrin subunit alpha 8	Plasma Membrane	other
KALRN	kalirin RhoGEF kinase	Cytoplasm	kinase
KCNA1	potassium voltage-gated channel subfamily A member 1	Plasma Membrane	ion channel
KCNA2	potassium voltage-gated channel subfamily A member 2	Plasma Membrane	ion channel
KCNC1	potassium voltage-gated channel subfamily C member 1	Plasma Membrane	ion channel
KCNC3	potassium voltage-gated channel subfamily C member 3	Plasma Membrane	ion channel
KCND3	potassium voltage-gated channel subfamily D member 3	Plasma Membrane	ion channel
KCNIP3	potassium voltage-gated channel interacting protein 3	Nucleus	transcription regulator
KCNMA1	potassium calcium-activated channel subfamily M alpha 1	Plasma Membrane	ion channel
LRP4	LDL receptor related protein 4	Extracellular Space	other
NFASC	neurofascin	Plasma Membrane	other
NOS2	nitric oxide synthase 2	Cytoplasm	enzyme
OPRD1	opioid receptor delta 1	Plasma Membrane	G-protein coupled receptor
P2RX2	purinergic receptor P2X 2	Plasma Membrane	ion channel
PLCB2	phospholipase C beta 2	Cytoplasm	enzyme
PTCH1	patched 1	Plasma Membrane	transmembrane receptor
RAB3B	RAB3B, member RAS oncogene family	Cytoplasm	enzyme
RASGRF1	Ras protein specific guanine nucleotide releasing factor 1	Cytoplasm	other
RHOA	ras homolog family member A	Cytoplasm	enzyme
SCN1B	sodium voltage-gated channel beta subunit 1	Plasma Membrane	ion channel
SCN2A	sodium voltage-gated channel alpha subunit 2	Plasma Membrane	ion channel
SCN5A	sodium voltage-gated channel alpha subunit 5	Plasma Membrane	ion channel
SGCB	sarcoglycan beta	Plasma Membrane	other
SGK1	serum/glucocorticoid regulated kinase 1	Cytoplasm	kinase
SHISA7	shisa family member 7	Plasma Membrane	other

SLIT2	slit guidance ligand 2	Extracellular Space	other
SNAP25	synaptosome associated protein 25	Plasma Membrane	transporter
SPARC	secreted protein acidic and cysteine rich	Extracellular Space	other
STX1B	syntaxin 1B	Plasma Membrane	other
SULF2	sulfatase 2	Plasma Membrane	enzyme
SV2A	synaptic vesicle glycoprotein 2A	Cytoplasm	transporter
SYT2	synaptotagmin 2	Cytoplasm	transporter
SYT6	synaptotagmin 6	Cytoplasm	transporter
THBD	thrombomodulin	Plasma Membrane	transmembrane receptor
VCAN	versican	Extracellular Space	other
YWHAB	tyrosine 3-monooxygenase/tryptophan 5-monooxygenase activation protein	Cytoplasm	transcription regulator
