# Supplementary material for: CYP2J2 Modulates Diverse Transcriptional Programs in Adult Human Cardiomyocytes
Source: Sci Rep. 2020 Mar 24;10:5329. doi: 10.1038/s41598-020-62174-w (PMC7093536; doi:10.1038/s41598-020-62174-w)
Supplement: Supplementary file 5 — Supplementary table S5 [file 41598_2020_62174_MOESM5_ESM.pdf]

Table S5. List of the most significant upstream regulators predicted to be activated in CYP2J2-silenced cardiomyoctes.

Upstream Regulator	Molecule Type	Predicted Activation State	Activation z-score	P-value
HIF1A	transcription regulator	Activated	3.731	1.22E-06
SHH	peptidase	Activated	2.393	2.93E-06
TGFB1	growth factor	Activated	2.029	4.91E-06
EDN1	cytokine	Activated	2.129	7.23E-06
CTNNB1	transcription regulator	Activated	3.73	7.89E-06
